# *WNT3* hypomethylation counteracts low activity of the Wnt signaling pathway in the placenta of preeclampsia

**DOI:** 10.1007/s00018-021-03941-4

**Published:** 2021-10-04

**Authors:** Linlin Zhang, Min Sang, Ying Li, Yingying Li, Erfeng Yuan, Lijun Yang, Wenli Shi, Yangyang Yuan, Bo Yang, Peifeng Yang, Enwu Yuan

**Affiliations:** 1 Department of Laboratory Medicine, Third Affiliated Hospital of Zhengzhou University, 7 Kangfu Qian Street, Zhengzhou, 450052 Henan People’s Republic of China; 2 Department of Obstetrics and Gynecology, Third Affiliated Hospital of Zhengzhou University, 7 Kangfu Qian Street, Zhengzhou, 450052 Henan People’s Republic of China; 3 Medical Research Centre, Third Affiliated Hospital of Zhengzhou University, 7 Kangfu Qian Street, Zhengzhou, 450052 Henan People’s Republic of China; 4 Marshall Medical Research Centre, Fifth Affiliated Hospital of Zhengzhou University, 3 Kangfu Qian Street, Zhengzhou, 450052 Henan People’s Republic of China

**Keywords:** Preeclampsia, Placentas, *WNT3* gene, DNA methylation, Preterm birth

## Abstract

Preeclampsia is a hypertensive disorder of pregnancy. Many studies have shown that epigenetic mechanisms may play a role in preeclampsia. Moreover, our previous study indicated that the differentially methylated genes in preeclampsia were enriched in the Wnt/β-catenin signaling pathway. This study aimed to identify differentially methylated Wnt/β-catenin signaling pathway genes in the preeclamptic placenta and to study the roles of these genes in trophoblast cells in vitro. Using an Illumina Infinium HumanMethylation 850 K BeadChip, we found that the Wnt signaling pathway was globally hypermethylated in the preeclamptic group compared with the term birth group, but hypomethylated in the preeclamptic group compared with the preterm birth group. Among all Wnt/β-catenin signaling pathway factors, *WNT3* was the most significantly differentially expressed gene and was hypomethylated in the preeclamptic group compared to the nonhypertensive groups, namely, the preterm birth group and term birth group. This result was confirmed by pyrosequencing. Through quantitative real-time PCR and western blot analysis, the *WNT3* gene was found to be highly expressed in preeclamptic placental tissues, in contrast to other WNT factors, which were previously reported to be expressed at low levels in placental tissues. Additionally, in the HTR8/SVneo cell line, knockdown of *WNT3* suppressed the Wnt/β-catenin signaling pathway, consistent with the findings for other WNT factors. These results prompted us to speculate that the *WNT3* gene counteracts the low activation state of the Wnt signaling pathway in the preeclamptic placenta through methylation modification.

**Supplementary Information:**

The online version contains supplementary material available at 10.1007/s00018-021-03941-4.

## Introduction

Preeclampsia (PE) is a common gestational complication that is characterized by hypertension, proteinuria, and other systemic disorders, and affects approximately 2–8% of pregnant women worldwide. It is the second leading cause of maternal death and can lead to serious maternal complications, including stroke, eclampsia, placental abruption, disseminated intravascular coagulation, and organ failure [1]. PE is also associated with adverse perinatal risks such as intrauterine growth restriction, low birth weight, and stillbirth [2]. Although many mechanisms of PE have been proposed, the etiology and pathogenesis of PE remain unclear. It is generally believed that insufficient trophoblast invasion leading to placental dysplasia plays a significant role in the development of PE [3]. Normal placental development is crucial during pregnancy, and multiple signaling pathways, including the Wnt signaling pathway, have been reported to be involved in regulating the proliferation, differentiation, and apoptosis of trophoblasts [4, 5]. Many recent studies have shown that epigenetic mechanisms may play a role in PE. Additionally, our previous study confirmed that the methylation levels in preeclamptic placental tissues were altered compared to those in control tissues and that the differentially methylated genes were significantly enriched in the Wnt signaling pathway [6].

**Fig. 1:**
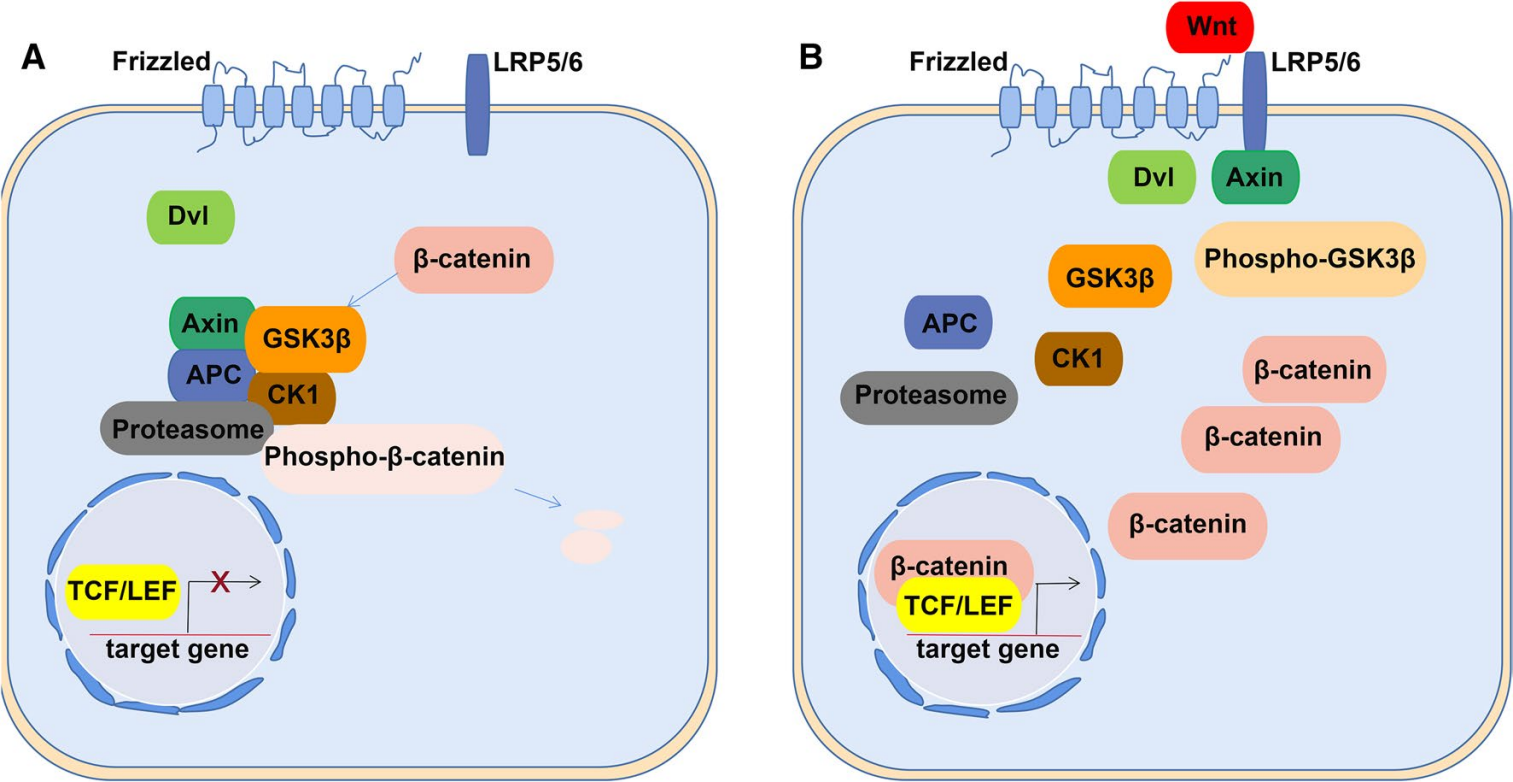
The canonical Wnt/β-catenin pathway. **A** Without Wnt ligand, β-catenin in the cytoplasm is phosphorylated and then degraded by ubiquitination. **B** With Wnt ligand, phosphorylated GSK3β and β-catenin increase in the cytoplasm, and the latter enters the nucleus to regulate gene expression. *LRP* lipoprotein receptor-related protein, *Dvl* disheveled, *APC* adenomatous polyposis coli, *GSK3β* glycogen synthase kinase 3β, *CK1* casein kinase 1, *TCT/LEF* T-cell factor/lymphocyte enhancer binding factor

The Wnt signaling pathway is an essential pathway in the regulation of cell proliferation, migration, and death in humans, and numerous studies have shown that the Wnt signaling pathway is involved in the development of many diseases and conditions, such as birth defects, cancers, and PE [7–9]. There are three Wnt signaling pathways: the canonical Wnt/β-catenin pathway, the noncanonical Wnt/Ca^2+^ pathway, and the Wnt/planar cell polarity (PCP) pathway [10]. Abnormal activation of the canonical Wnt/β-catenin pathway plays an important role in the pathogenesis of various human diseases [11]. The Wnt/β-catenin signaling pathway comprises 19 ligands, 10 membrane receptors, and many transcription factors and inhibitors [12], each of which mediates a different cellular function. β-Catenin is an important link in the Wnt/β-catenin signaling pathway. In the absence of Wnt signaling, cytoplasmic β-catenin is phosphorylated by active (non-phosphorylated) glycogen synthase kinase 3β (GSK3β) and is then degraded through the ubiquitin–proteasome pathway [13]. In the presence of Wnt ligands, the receptors Frizzled and lipoprotein receptor-related protein 5/6 (LRP5/6) recruit Disheveled (Dvl) and Axin proteins in the cytoplasm, and GSK3β is phosphorylated. Then, active (non-phosphorylated) β-catenin accumulates in the cytoplasm and enters the nucleus to interact with members of the T-cell factor/lymphocyte enhancer factor (TCF/LEF) family of transcription factors and regulate the expression of downstream target genes [14–16] (Fig. 1).

Many factors have been reported to be abnormally expressed in preeclamptic placental tissues, and their expression levels can be regulated by DNA methylation. The expression of *WNT1*, *WNT2*, *WNT2b*, *WNT5a,* and *β-catenin* was shown to be decreased in preeclamptic placental tissues, while the expression of inhibitors such as *DKK1*, *WIF1,* and *SFRP4* was increased [17–20]. Moreover, in our previous study, we found that *WNT2* was hypermethylated and downregulated and that *DKK1* was hypomethylated and overexpressed in early onset PE [17]. Analysis of the differentially methylated genes in the Wnt/β-catenin pathway will improve our understanding of the pathogenesis of PE and provide valuable information for researchers and clinicians.

The purpose of this study was to analyze the methylation levels of Wnt/β-catenin signaling pathway genes, to verify the differentially methylated genes and their expression levels in placentas, and to study the roles of these genes in trophoblast cells in vitro*,* thus possibly revealing the involvement of candidate genes in the pathogenesis of PE.

## Materials and methods

### Study subjects

Subjects were recruited from the Third Affiliated Hospital of Zhengzhou University from August 2017 to February 2019 and were divided into three groups: early onset PE (PE, *n* = 30), preterm birth (PB, *n* = 30), and term birth (TB, *n* = 30). Early onset PE was diagnosed according to the criteria of the American Congress of Obstetricians and Gynecologists [21]. Women who delivered at or after 37 weeks of gestation without complications during pregnancy were recruited as the TB group. Women with spontaneous preterm delivery between 28 weeks + 0 days and 36 weeks + 6 days of gestation were defined as the PB group. The etiologies included premature membrane rupture, oligohydramnios, and cervical incompetence. Pregnant women with chronic hypertension, diabetes mellitus, renal disease, fetal malformations, or multiple pregnancies were excluded from the study. All participants signed informed consent forms. The study was approved by the Human Ethics Committee of the Third Affiliated Hospital of Zhengzhou University. The procedures used in this study adhere to the tenets of the Declaration of Helsinki.

### Sample collection

Placental tissues (5 × 5 × 5 mm) were obtained within 15 min after delivery, avoiding necrotic, infarcted, and calcified areas. Then, the tissues were washed with cold PBS to remove maternal and fetal blood. The placental tissues used for RNA extraction were stored in an RNA bank (CWBIO, China). Placental tissues were snap frozen in liquid nitrogen for 10 min and were then stored at − 80 °C until use.

### Genomic DNA methylation profiling

Genomic DNA was extracted with a DNeasy Blood & Tissue Kit (Qiagen, Germany). The concentration and purity of the DNA were determined with a NanoDrop 2000 spectrophotometer (Thermo Fisher Scientific). A260/A280 is between 1.8 and 2.0, and A260/A230 is greater than 2.0. Then, DNA was bisulfide converted with a Qiagen EpiTect Bisulfite Kit (Qiagen, Germany). Illumina Infinium HumanMethylation850 K BeadChip (Illumina Inc, USA) was used to assess genome-wide DNA methylation according to the manufacturer’s standard protocol. Differential gene expression was determined using unpaired *t* test, implemented in the R (version 2.14.0) package. CpG sites |∆*β*|≥ 0.10 (test group vs. control group) and *P* < 0.05 was considered as differentially methylated site (DMSs). Gene ontology (GO, http://www.geneontology.org) and Kyoto encyclopedia of genes and genomes (KEGG, http://www.kegg.jp/) pathway enrichment analysis are used to clarify the function and biological pathways of differentially expressed methylation sites from our data.

### Pyrosequencing

Genomic DNA was extracted with a DNeasy Blood & Tissue Kit (Qiagen, Germany). DNA was bisulfide converted with a Qiagen EpiTect Bisulfite Kit (Qiagen, Germany). Primers were designed with PyroMark Assay Design 2.0 and run in a Qiagen PyroMark Q96 MD (Qiagen). According to our previous study on the methylation levels of PE using an Illumina Infinium HumanMethylation850K BeadChip, the identified differential sequences of *WNT3* were verified by pyrosequencing. The sequences contained 4 CpG sites: A**CG**G**CG**GG**CG**TTTTA**CG**AGGTGAGGGTTATGGTTGAAGGAA. The sequences of the *WNT3* primers were as follows: forward primer, TTGTGTAGGGAATTGTGGTAG; reverse primer, ACCAAAAATATCTAACCCCCTAAC; sequencing primer, AGTTTTATAGAGGTTTGGA. The reverse primer was labeled with biotin at the 5’ end.

### RNA extraction and quantitative real-time PCR

RNA was extracted from 90 placental tissues and cells with TRIzol Reagent (CWBIO, China). The concentration and purity of the RNA were determined with a NanoDrop 2000 spectrophotometer (Thermo Fisher Scientific), and ethidium bromide staining of nucleic acids before agarose gel electrophoresis was used to evaluate the RNA integrity. Less than 1 μg of mRNA was reverse transcribed into cDNA with ReverTra Ace® qPCR RT Master Mix (TOYOBO, Japan). The sequences of the *WNT3* primers were as follows: forward primer, TTCGGCGTGTTAGTGTCCAG; reverse primer, AGGCGCTGTCATACTTGTCC. The sequences of the *GAPDH* primers were as follows: forward primer, AGAACGGGAAGCTTGTCATC; reverse primer, CATCGCCCCACTTGATTTTG. The relative mRNA expression levels of the genes were evaluated using the 2^−ΔΔ*C*t^ method.

### Immunohistochemistry (IHC)

Immunohistochemical staining was used to evaluate the location and expression of WNT3 proteins in placental tissues. Placental tissue sections were heated for 2 h at 60 °C, deparaffinized by immersion in xylene two times for 10 min each, and dehydrated through a series of graded ethanol solutions (100, 95, 85 and 75%).

Sections were immersed in the prepared antigen retrieval solution and were then sequentially heated to greater than 90 °C in a microwave oven and then quenched at a low temperature for 20 min. Furthermore, the sections were incubated with an anti-Wnt3 antibody (1:200; ab116222; Abcam) at 4 °C overnight. After washing with PBS three times, the sections were incubated with a biotin-conjugated secondary antibody (1:200; OriGene Technologies, Inc.) for 1 h at room temperature. The sections were stained with DAB reagent, counterstained with hematoxylin, and finally sealed with neutral balsam. The slides were examined by inverted fluorescence microscopy (OLYMPUS IX-71, Tokyo, Japan). The staining of the sections was independently evaluated by two pathologists.

### Western blotting analysis

RIPA lysis buffer containing a protease inhibitor (CWBIO, China) was used to extract protein from placental tissues and cells. The total protein concentration was measured with a BCA assay kit (Thermo Fisher Scientific, Inc.). For western blot analysis, approximately 40 μg of protein was separated on an 8% SDS-PAGE gel and electrophoretically transferred to PVDF membranes. The membranes were blocked for 2 h at room temperature with blocking buffer (5% nonfat milk, 0.1% Tween 20). Then, the membranes were incubated at 4 °C overnight with anti-Wnt3 (1:1000; ab116222; Abcam), anti-β-catenin (1:1000; Cell Signaling Technology, Inc.), anti-phospho-β-catenin (1:1000; Cell Signaling Technology, Inc.), anti-GSK3β (1:1000; Cell Signaling Technology, Inc.), anti-phospho-GSK3β (1:1000; Cell Signaling Technology, Inc.), and anti-β-actin (1:2000; ab8227; Abcam) antibodies separately. The membranes were then incubated with fluorescent secondary antibodies (1:15,000; LI-COR, USA) for 2 h at room temperature. An infrared laser scanning imaging system (Odyssey CIX, LI-COR, USA) was used to determine the fluorescence intensity.

### Cell culture and treatment

HTR8/SVneo and JAR cells, which were provided by American Type Culture Collection (USA), were cultured in high-glucose DMEM (HyClone; GE Healthcare Life Sciences) containing 10% fetal bovine serum (FBS), 100 U/ml ampicillin, and 100 U/ml streptomycin at 37 °C in humidified incubators with 5% CO_2_. After adherence, the next day, the culture medium was changed to fresh medium-containing 5 μM 5-aza-2'-deoxycytidine (5-aza-dC; A3656, Sigma, USA) and was replaced daily thereafter. The control group was treated with 0.1‰ DMSO.

### Cell transfection

Plasmids were obtained from GeneCopoeia (USA). ShRNA oligos were ligated into the pUC Ori-shRNA-CMV expression vector containing the U6 promoter (GeneCopoeia, USA). Transfection was performed according to the manufacturer’s protocols (Invitrogen; Thermo Fisher Scientific). Cells were divided into three groups: The control group (untreated), shRNA-*WNT3* group (transfected with *WNT3*-shRNA), and sh-negative control group (transfected with an unrelated sequence). Transfection was performed at a cell confluence of approximately 60% (approximately 14 h after seeding), and 5 µl of shRNAs were transfected into cells using 5 µl of Lipofectamine 3000 reagent (Invitrogen; Thermo Fisher Scientific). The transfection efficiency was assessed 48 h after transfection.

### Cell proliferation assay

Cells were inoculated into 96-well plates (1000–5000 cells/well), and a Cell Counting Kit-8 (CCK-8; Dojindo Molecular Technologies, Inc.) assay was used to determine cell viability. The inoculated cells were placed in a 37 °C incubator for 2–4 h to allow adherence to the plate walls. Subsequently, 10 µl of CCK-8 solution was added to each well and incubated at 37 °C for 1 h. Absorption values were obtained using a microplate reader (Bio-Rad Laboratories, Inc.) at 450 nm.

### Statistical analysis

SPSS (Version 25.0, IBM, New York, USA) and GraphPad Prism (Version 8.4.2, Inc, San Diego, CA, USA) were used for statistical analysis. The quantitative data were expressed as the mean ± standard deviation values. One-way ANOVA or the Kruskal–Wallis test was performed to compare data among the three groups, and Student’s *t* test or the Mann–Whitney test was performed to compare data between two groups. Welch’s ANOVA and Welch's *t* tests were used for correction when variance was inconsistent. Pearson correlation analysis was performed to analyze the relationship of two continuous variables. *R*^2^ represents the contribution of an independent variable to the regression relationship, and r is defined as the correlation coefficient. The immunohistochemical staining intensity of the WNT3 protein was determined using the Chi-square test. The Kruskal–Wallis test with the Bonferroni correction was used to analyze differences among multiple groups. *P < 0.05, **P < 0.01. ***P < 0.001, ****P < 0.0001.

## Results

### Clinical characteristics of the study subjects

The clinical characteristics of the recruited women were shown in Table 1. The mean gestational age at delivery in the PE and PB groups was significantly lower than that in the TB group (*P* < 0.01), but did not differ significantly between the PE and PB groups. The systolic and diastolic blood pressures in the PE group were significantly higher than those in the PB and TB groups (*P* < 0.01). The neonatal birth weight in the PE group was significantly lower than that in the PB group, and proteinuria was significantly more severe in the PE group than in the other two groups (*P* < 0.01). The maternal age and fetal sex did not differ significantly among the three groups.

**Table 1:** The clinical characteristics of the three groups

Characteristics	TB (*n* = 30)	PB (*n* = 30)	PE (*n* = 30)
Maternal age (years)	32.45 ± 4.29	31.33 ± 4.95	32.61 ± 5.04
Gestational age (weeks)	39.09 ± 0.72	34.37 ± 1.91^a^	33.12 ± 1.92^a^
Systolic pressure (mmHg)	117.68 ± 10.06	115.93 ± 10.12	168.16 ± 18.42^ab^
Diastolic pressure (mmHg)	75.63 ± 10.13	71.44 ± 8.11	102.36 ± 8.48^ab^
Proteinuria (g/24 h)	0.205 ± 0.130	0.134 ± 0.025	3.567 ± 0.651^ab^
Neonatal birth weight (g)	3505.54 ± 396.74	2296.30 ± 509.52^a^	1711.48 ± 499.87^ab^
Fetal gender (male/female)	18/12	13/17	15/15

*TB* term birth, *PB* preterm birth, *PE* preeclampsia

^a^Compared with TB group, *P* < 0.05

^b^Compared with PB group, *P* < 0.05. Unpaired t test

### DNA methylation levels in the Wnt signaling pathway are altered in PE

**Fig. 2:**
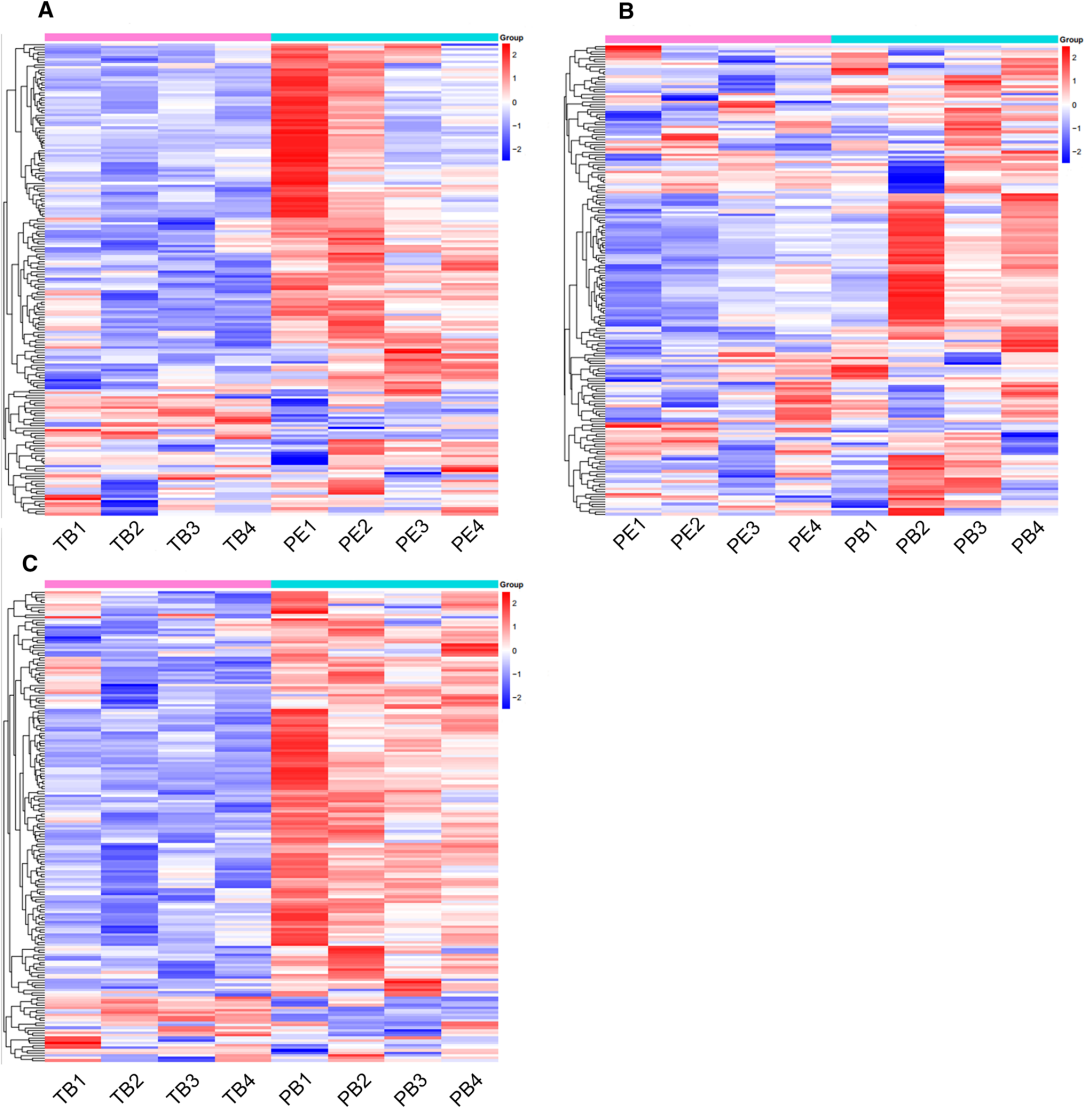
The global methylation level of Wnt signaling pathway among three groups detected by Illumina 850 K Beadchip. **A** Comparison between TB and PE; **B** Comparison between PE and PB; **C** Comparison between TB and PB. Blue color represents hypomethylated sites, while red color represents hypermethylated sites.* TB* term birth, *PB* preterm birth, *PE* preeclampsia. *β* value represents the methylation levels of detected sites

The distribution of probe sites in all ligands in the Wnt/β-catenin signaling pathway with the Illumina Infinium HumanMethylation 850 K BeadChip was shown in supplemental Fig. 1. Then, we mapped the methylation levels at detected sites in all ligands in the Wnt/β-catenin signaling pathway (Fig. 2). The Wnt/β-catenin signaling pathway was globally hypomethylated in the PE group compared to the PB group but hypermethylated in the PE group compared with the TB group.

*WNT3*, *WNT5b*, *WNT11*, *SFRP2,* and *SFRP5* displayed the greatest differences in methylation levels according to the 850 K BeadChip analysis results (∆β ˃ 5%) (Table 2). We then analyzed these genes for differential methylation sites (Supplemental Fig. 2 and Supplemental Table 1). Finally, based on data consistency and the significance of the differences, *WNT3* was selected for functional study in trophoblast cell lines.

**Table 2:** The Wnt signaling pathway factors with significant difference in methylation levels

∆β	> 0%	> 5%
PE vs TB	*WNT2b*, *WNT4*, *WNT5b*, *WNT6*, *WNT7b*, *WNT8b*, *WNT9a*, *WNT9b*, *WNT10b*, *WNT11*, *WNT16*, *DKK1*, *DKK3*, *SFRP1*, *WIF1*	*SFRP2*, *SFRP5*
PB vs TB	*WNT2b*, *WNT4*, *WNT5b*, *WNT6*, *WNT7b*, *WNT8a*, *WNT8b*, *WNT9a*, *WNT9b*, *WNT10a*, *WNT10b*, *WNT16*, *DKK1*, *DKK3*, *SFRP1*, *WIF1*	*WNT11*, *SFRP2*
PE vs PB	*WNT2b*, *WNT9b*, *WNT11*, *WNT16*	*WNT3*, *WNT5b*

*β* value was used to represent the methylation levels of all the probes

∆*β* is the difference in *β* value between the two groups

*TB* term birth, *PB* preterm birth, *PE* preeclampsia

*P* < 0.05

### The DNA methylation level of the *WNT3* gene is decreased in the preeclamptic placenta

**Fig. 3:**
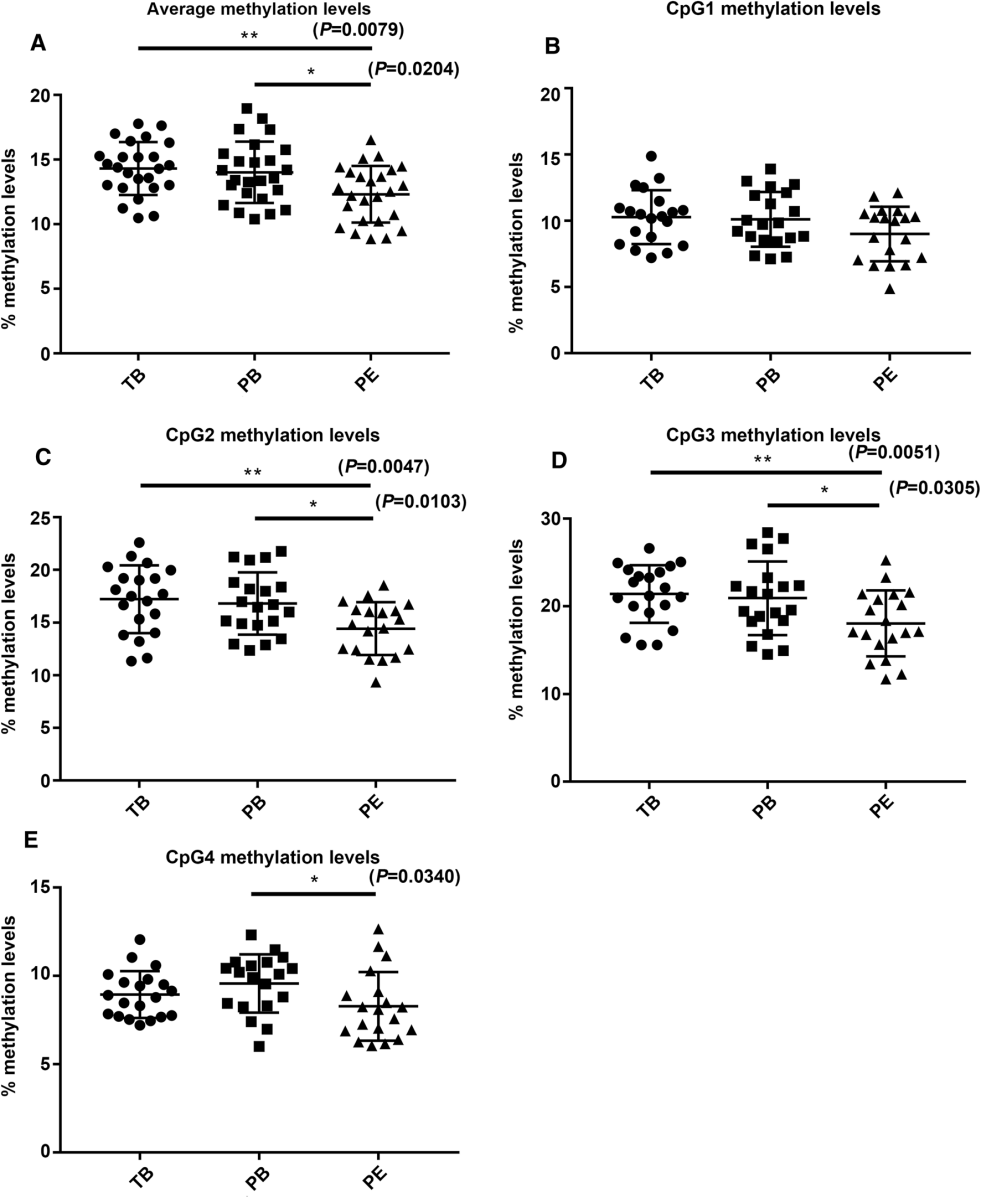
The methylation levels of *WNT3* gene by pyrosequencing. **A** The average levels of the four CpGs in PE (12.4% ± 2.2%) compared with TB (14.5% ± 2.2%) and PB (14.3% ± 2.5%) groups. **B**–**E** The methylation levels of the four CpGs.* TB* term birth, *PB* preterm birth, *PE* preeclampsia. Unpaired t test. *P < 0.05, **P < 0.01

Among the differentially methylated genes in the Wnt/β-catenin signaling pathway, *WNT3* was the most significantly differentially expressed gene in the PE group compared to the PB group. The promoter region of *WNT3* was hypomethylated in the PE group compared to the PB group, while there was no significant difference between the TB and PE groups. The change in the methylation level of the *WNT3* promoter was confirmed by pyrosequencing. The sequence of the methylation site CG24114556 with the most significant difference is A**CG**G**CG**GG**CG**TTTTA**CG**AGGTGAGGGTTATGGTTGAAGGAA (∆*β* ≥ 10% and *P* < 0.05) (Table 3). The methylation levels of the four CpGs and the mean methylation level were shown in Fig. 3. The promoter region of *WNT3* was significantly hypomethylated in the PE group compared to the other two groups. In the PE group, the methylation levels of CpG2, CpG3, and CpG4 were significantly reduced compared with those in the other two groups, while there was no significant difference in CpG1 among the three groups.

**Table 3:** The differential methylated sites of *WNT3* gene between PE and PB

DMS	Gene	∆*β* (%)	Location
cg24114556	*WNT3*	10%	TSS1500
cg16340130	*WNT3*	6%	Gene body
cg02721983	*WNT3*	3%	Gene body

*DMS* differential methylated site, *TSS* transcription started site

*P* < 0.05

### *WNT3* mRNA and protein expression are upregulated in the preeclamptic placenta

**Fig. 4:**
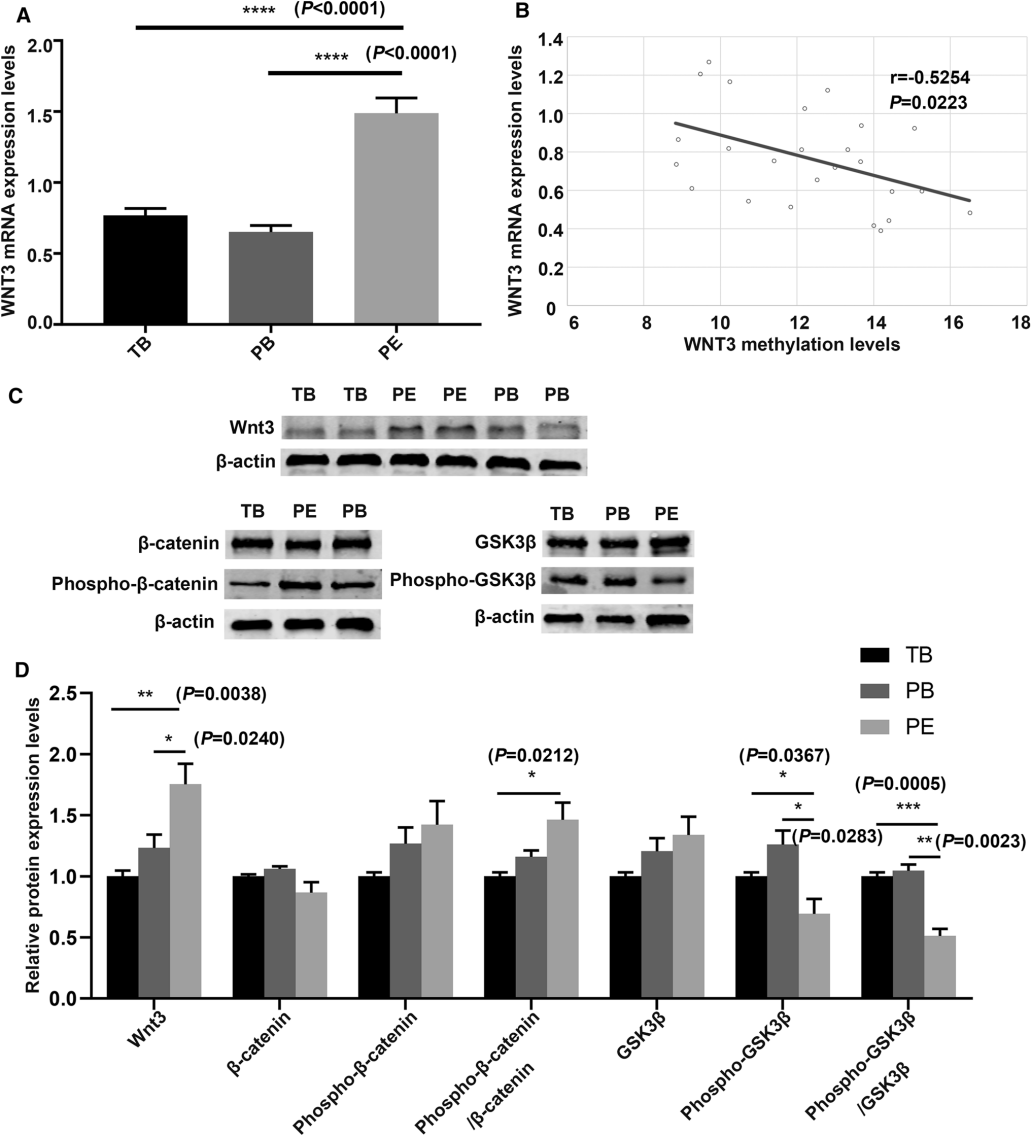
The expression levels of *WNT3* gene in placentas of the three group and its correlation with methylation levels. **A** The mRNA expression levels of *WNT3* in placentas in the three groups. **B** The correlation between mRNA expression and methylation levels. *r* = − 0.525, *R*^2^ = 0.2071, *P* < 0.05. **C**, **D** The relative protein expression of *WNT3* in placentas of the three groups by western blotting.* TB* term birth, *PB* preterm birth, *PE* preeclampsia. Unpaired *t* test, Welch's *t* test, Mann–Whitney test, and Pearson correlation analysis. *P < 0.05, **P < 0.01. ***P < 0.001, ****P < 0.0001

**Fig. 5:**
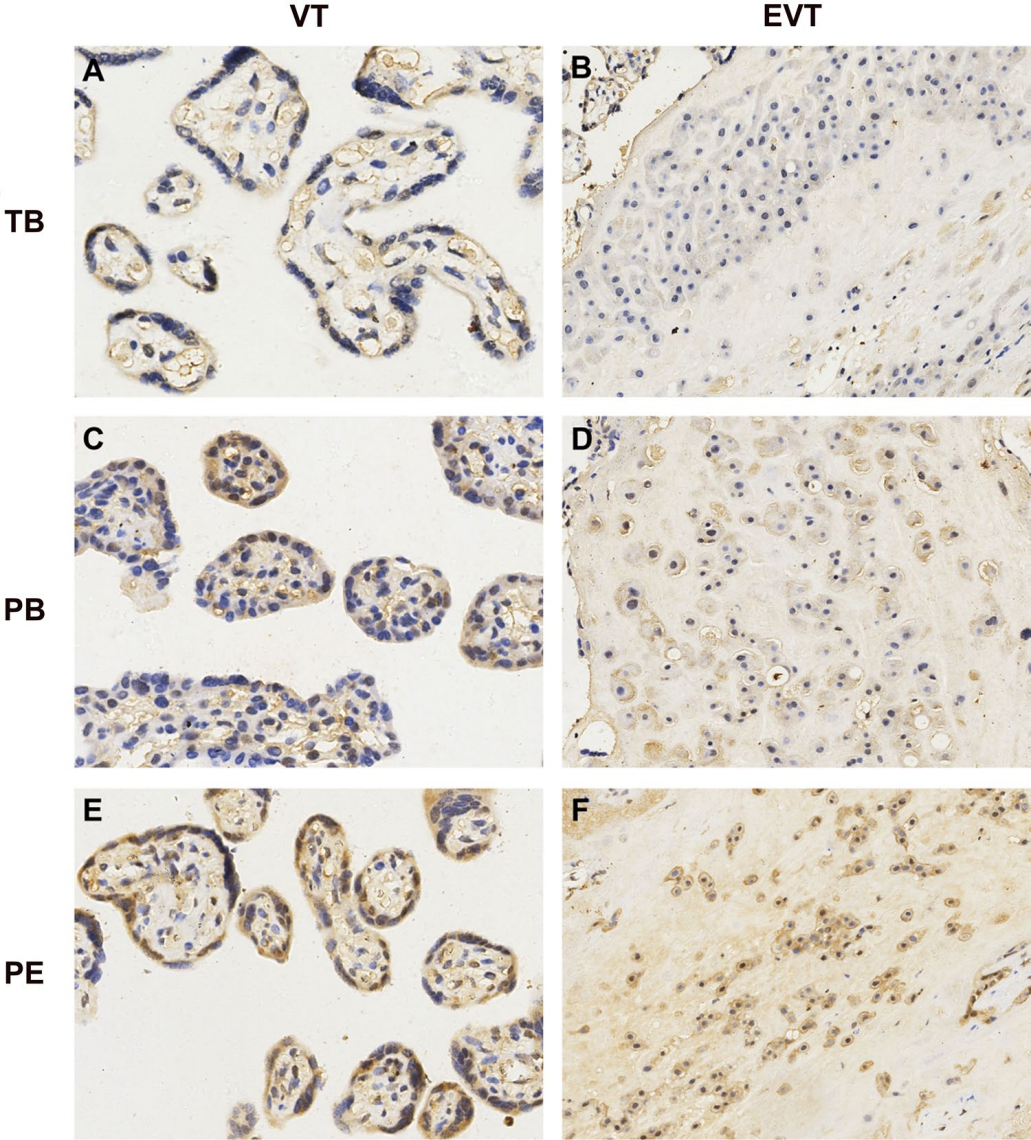
Immunostaining of WNT3 protein in placental tissue sections of the three groups WNT3 protein was located in the villous trophoblasts (VT) and extravillous trophoblasts (EVT). *TB* term birth, *PB* preterm birth, *PE* preeclampsia. Original magnification 400 × for **A**, **C**, **E**, 200 × for **B**, **D** and **F**

We next examined the mRNA and protein levels of *WNT3* and analyzed the relationship of these levels with its methylation level. The mRNA expression level of *WNT3* was shown in Fig. 4A. The mRNA expression level of *WNT3* in the PE group was significantly higher than that in the other two groups. We analyzed the correlation between the methylation level and mRNA expression level of *WNT3* (Fig. 4B), and found that the expression level of *WNT3* mRNA increased as its DNA methylation level decreased. The mRNA expression level of *WNT3* was negatively correlated with its DNA methylation level (*r* = − 0.525, *R*^2^ = 0.2071, *P* < 0.05). While, there was no correlation between the mRNA expression level of *WNT3* and methylation level with donor age (Supplemental Fig. 3). The protein expression level of *WNT3* in placental tissues was evaluated by western blotting (Fig. 4C, D) and was found to be significantly higher in preeclamptic placentas than in the placentas of the other two groups. Immunohistochemical staining showed that the WNT3 protein was localized in villous trophoblasts (VTs) and extravillous trophoblasts (EVTs) (Fig. 5). In preeclamptic placentas, the WNT3 protein levels in both VTs and EVTs were higher than those in the other two groups. The level of phosphorylated β-catenin in preeclamptic placentas was increased, which indicated that the activity of the Wnt/β-catenin signaling pathway was decreased.

### Demethylation treatment increases *WNT3* expression

**Fig. 6:**
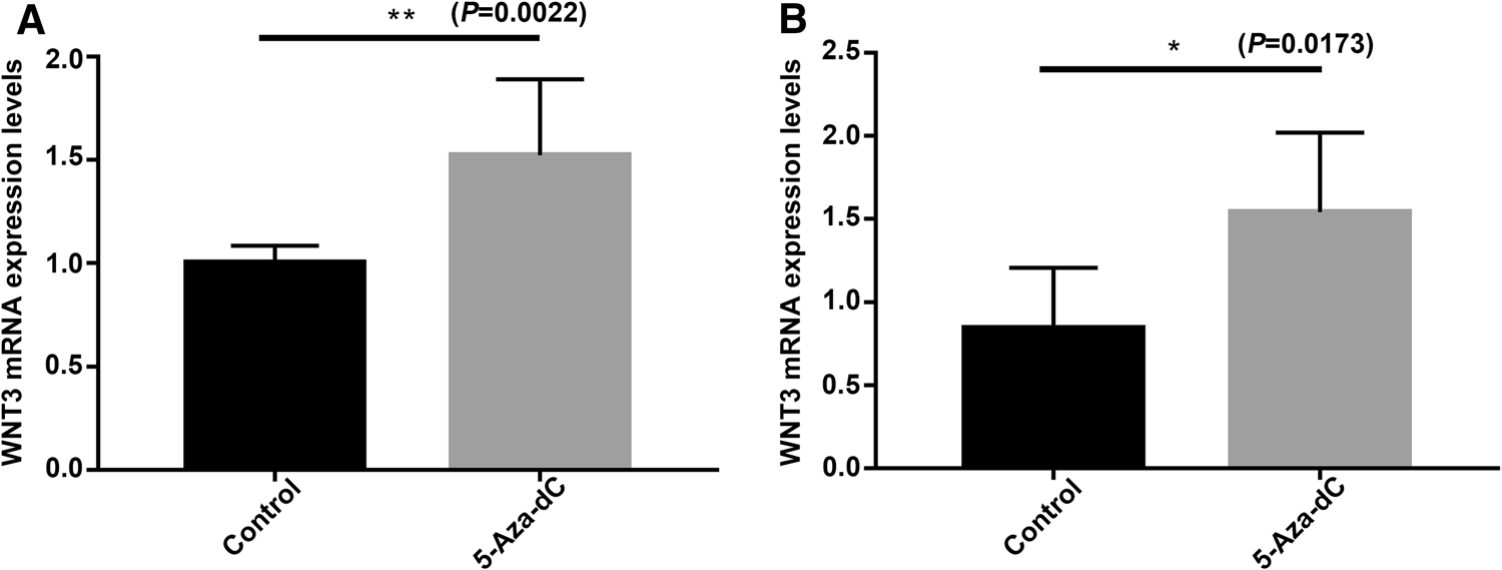
The mRNA expression levels of *WNT3* after demethylation by 5-Aza-dC in JAR and HTR8/Svneo cell lines. **A** The expression of *WNT3* in JAR cell line. **B** The expression of *WNT3* in HTR8/Svneo cell line. 5-Aza-dC: 5-Aza-2'-deoxycytidine. Unpaired *t* test and Mann–Whitney test. *P < 0.05; **P < 0.01

According to our study on placental tissues, *WNT3* was hypomethylated and its expression level was increased in PE. We used a human first-trimester EVT cell line and a human choriocarcinoma cell line to further analyze whether changes in *WNT3* expression are due to changes in its methylation status. In both cell lines, treatment with 5-aza-dC, which decreases the methylation level, caused a significant increase in *WNT3* expression (*P* < 0.05) (Fig. 6).

### Silencing the *WNT3* gene decreases trophoblast cell proliferation

**Fig. 7:**
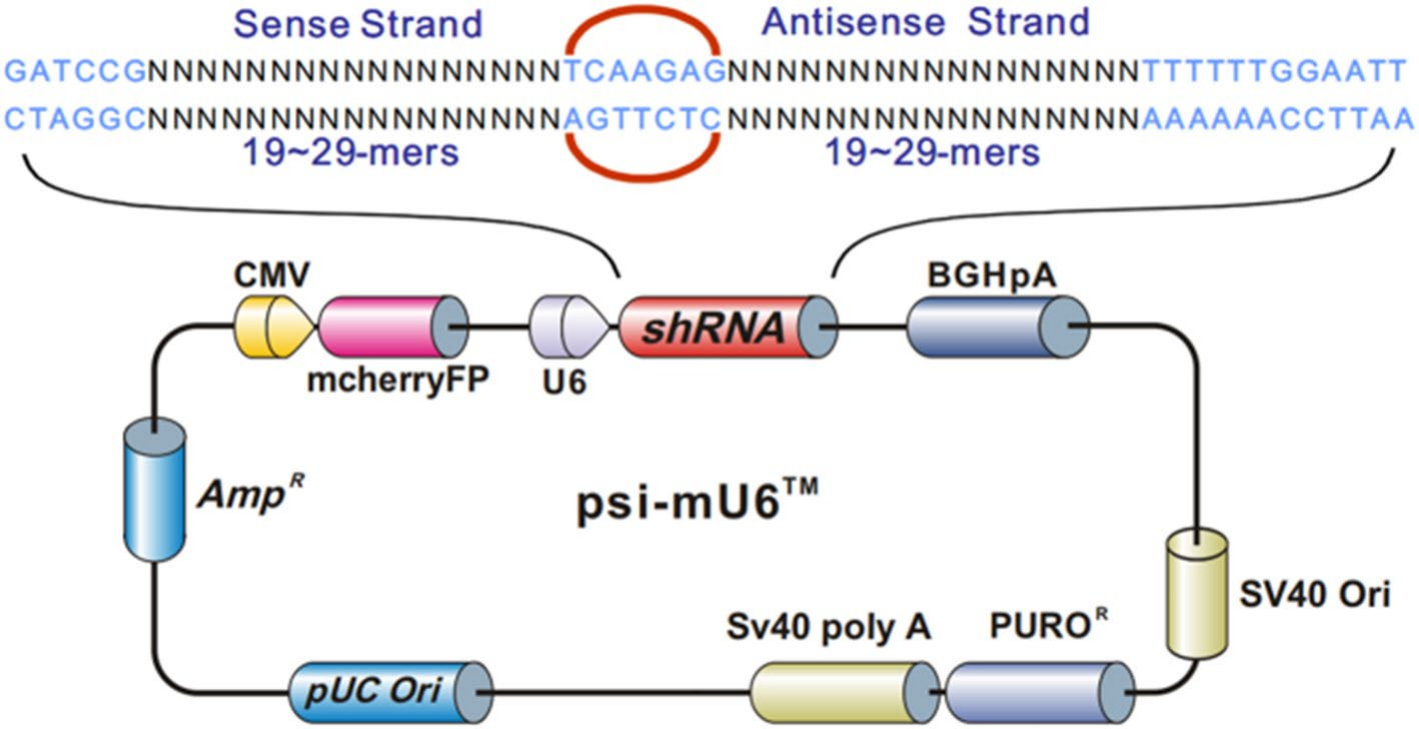
The shRNA sequence of *WNT3* gene

**Fig. 8:**
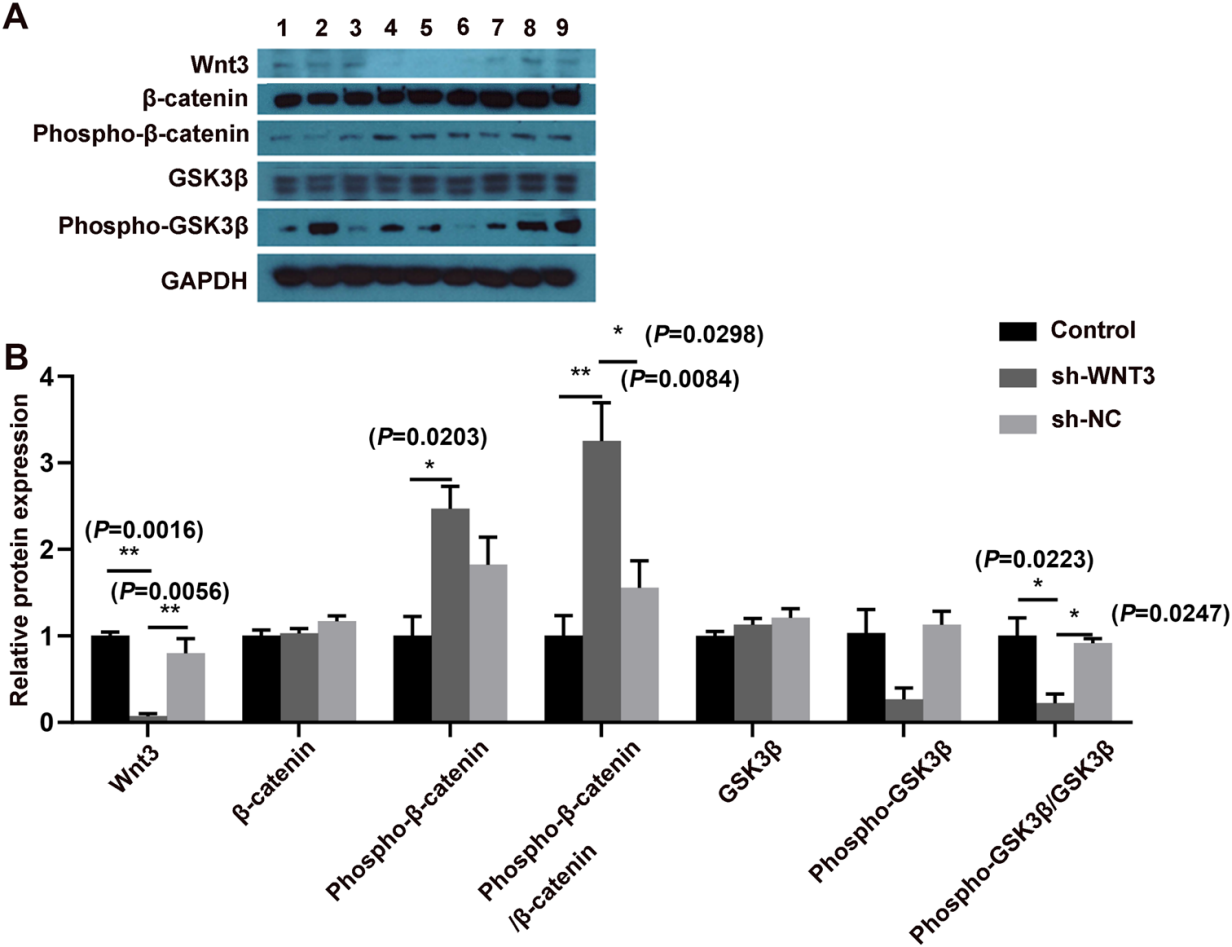
Loss of *WNT3* suppressed the Wnt/β-catenin signaling pathway. **A** Expression of Wnt/β-catenin signaling pathway proteins in HTR8/SVneo cell line after transfection with shRNA. 1–3, Control; 4–6, sh-*WNT3* (cells were transfected with shRNA-*WNT3*); 7–9, sh-NC. **B** The relative protein expression of WNT3, β-catenin, phosphorylated β-catenin, GSK3β, phosphorylated GSK3β, and ratio of phosphorylated and non-phosphorylated protein in HTR8/SVneo cell line. One-Way ANOVA. *P < 0.05, **P < 0.01, ***P < 0.001

**Fig. 9:**
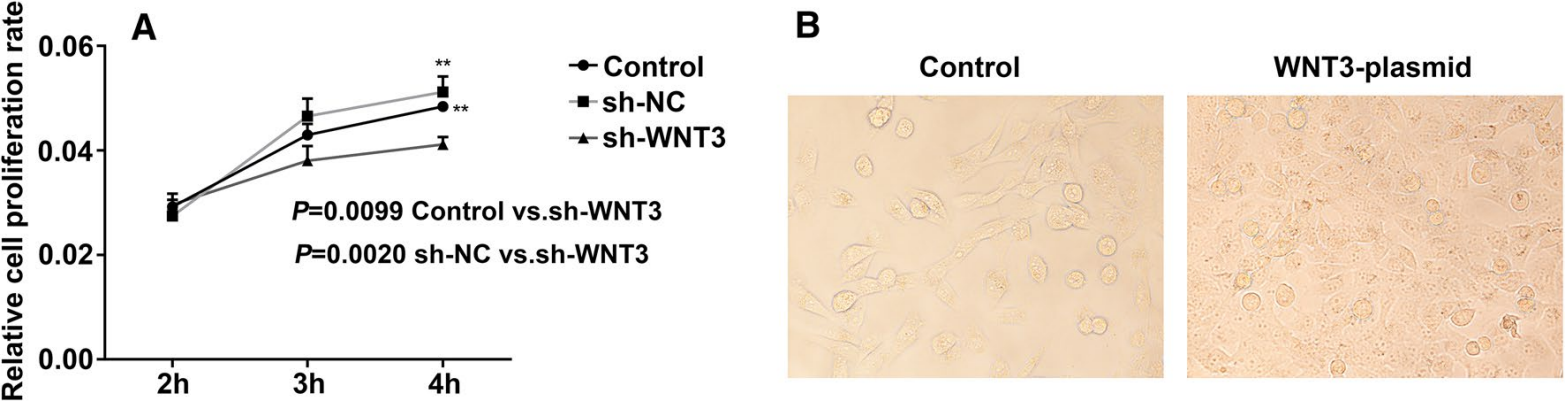
*WNT3* promoted trophoblast cell proliferation. **A** Cell Counting Kit-8 assay was used to detect the proliferation of HTR8/SVneo cells. **B** Cell proliferation increased after transfection with *WNT3*-plasmid in HTR8/SVneo cells. One-way ANOVA. *P < 0.05, **P < 0.01. Image acquisition tools: IBM SPSS Statistics 25 and GraphPad Prism 8.4.2. Image processing software packages: Adobe photoshop cs6 13.0.1

We transfected shRNA-*WNT3* (Fig. 7) into the HTR8/SVneo cell line to evaluate Wnt/β-catenin signaling pathway activity and assess cell viability and proliferation. As shown in Fig. 8, after silencing *WNT3*, the relative protein expression of *WNT3* and phosphorylation of GSK3β were decreased, and phosphorylation of β-catenin was increased after silencing *WNT3*, suggesting that low expression of *WNT3* suppresses the Wnt/β-catenin signaling pathway in PE. A CCK-8 assay was performed to evaluate trophoblast cell viability, and the results revealed that silencing *WNT3* decreased the viability and proliferation of HTR8/SVneo cells compared with control cells (Fig. 9a).

## Discussion

PE is a systemic disorder that involves multiple factors, complex mechanisms, and many signaling pathways. PE can progress rapidly to serious complications, including death of both the mother and fetus. The pathogenesis is still controversial, and several theories have been proposed. PE is recognized to be a disease of placental origin: the invasion ability of trophoblasts is decreased in early pregnancy, resulting in shallow placental implantation, which is the key link in the pathogenic mechanism [22].

Depending on the gestational week in which clinical symptoms appear, PE is classified as early-onset (< 34 weeks of gestation) or late-onset (≥ 34 weeks of gestation) PE. Studies have shown that the pathogenesis of early- and late-onset PE is different [23, 24]. Early-onset PE is likely to be a placenta-related disease and caused by insufficient perfusion of the spiral artery, while late-onset PE seems to be a manifestation of metabolic disorders. The condition of early-onset PE is exacerbated with increasing gestational week, and multiple organ failure may occur earlier than in late-onset PE. In the present study, the gestational age in the PE group was 33.12 ± 1.92 weeks, thus, the preeclamptic subjects enrolled in this study were considered early-onset PE patients.

DNA methylation, as a classical mechanism of epigenetic regulation, is widely involved in placental development and trophoblast differentiation. Many factors affect the level of DNA methylation in placental tissues, including gestational age, intrauterine environment, fetal sex, and so on [25, 26]. Several studies have indicated that among these factors, gestational age is an important factor affecting the level of DNA methylation in placental tissues. Novakovic et al. found significant differences in methylation levels among the first, second and third trimesters of pregnancy [27]. In the current study, the subjects were divided into three groups: the PE, PB, and TB groups. In most previous studies, the TB group was used as a control group, however, the large gestational age difference between the subjects in this group and subjects in the other groups can be an important factor affecting DNA methylation in placentas. Therefore, we added the PB group to eliminate the influence of gestational age, as there was no significant difference in gestational age between the PE and PB groups. Fetal sex is another important factor affecting gene methylation, and methylation alterations are more frequently observed in female placentas [26]. In our study, there was no significant difference in the fetal sex ratio among these three groups.

In the present study, we found that the global methylation level of the Wnt/β-catenin signaling pathway in the PE group was higher than that in the TB group and lower than that in the PB group (Fig. 2). The methylation levels of *WNT3*, *WNT5b*, *WNT11*, *SFRP2*, and *SFRP5* were the most different among the three groups of placental tissues (Table 2). To exclude the effect of gestational age, we selected significant differentially expressed genes with statistically in the PE and PB groups. Ultimately, *WNT3* was selected for functional study in trophoblast cell lines. Then, it was confirmed by pyrosequencing that the average methylation level of the *WNT3* promoter region in the PE group was lower than that in the other two groups (Fig. 3).

The *WNT3* gene is a member of the *WNT* gene family, and its encoded protein plays an important role in many diseases as a ligand of the Wnt/β-catenin signaling pathway. Studies have shown that *WNT3* is upregulated in human breast, rectal, lung, gastric, and hepatocellular cancer tissues and plays a key role in the occurrence and development of these tumors by activating the Wnt/β-catenin signaling pathway [28–34]. *WNT3* also plays an important role in embryonic development and regulates trophectoderm differentiation in blastocysts [35]. Kaloglu C et al. found that *WNT3* is involved in regulating decidualization, stromal cell proliferation, and trophoblast cell infiltration in the rat uterus [36]. In the present study, we found that the promoter region of *WNT3* was hypomethylated and that its expression was increased in preeclamptic placentas. Highly expressed WNT3 ligand binds to surface receptors on trophoblast cells to activate the Wnt/β-catenin signaling pathway. Western blot analysis of placental tissues indicated that the level of phosphorylated β-catenin was increased in preeclamptic placental tissue (Fig. 4C, D) and that Wnt/β-catenin signaling pathway activity was decreased in PE. Subsequently, we verified the function of *WNT3* in trophoblast cell lines in vitro. After silencing *WNT3*, the level of phosphorylated β-catenin in HTR8/SVneo cells was increased compared with that in the control group, and the Wnt/β-catenin signaling pathway was suppressed. In addition, the proliferation ability of trophoblast cells was reduced (Fig. 9a). In addition, after transfection of the *WNT3* overexpression plasmid into trophoblast cells, cell proliferation was increased (Fig. 9b). However, Pollheimer et al. found that *WNT3A* overexpression did not affect the proliferation of trophoblast cells [45]. Thus, more experiments are needed to explore the effect of *WNT3* overexpression on trophoblast proliferation. Some researchers have found similar phenomena in breast cancer cell lines, esophageal squamous cell carcinoma cell lines, and osteoarthritic chondrocyte cell lines, in which expression of nuclear β-catenin was decreased and the Wnt/β-catenin signaling pathway was suppressed after knockdown of *WNT3* [37–39]. Thus, *WNT3* may be an activator of the Wnt/β-catenin signaling pathway. Xing et al. found that knockdown of *WNT3* expression in tumor cells significantly blocked cell proliferation, delayed cell cycle progression, and suppressed cell invasion and metastasis, accompanied by increased apoptosis [31]. In addition, increasing the expression of *WNT3* accelerated the invasion and migration of trophoblast cells [40]. The results of this study revealed high expression of *WNT3.*

In the preeclamptic placenta, the high expression level of *WNT3* is inconsistent with the decrease in Wnt/β-catenin signaling pathway activity. The results of our previous studies indicated that the expression levels of *WNT1*, *WNT2*, and *WNT2B* in preeclamptic placental tissue are decreased [4, 7, 17, 19]. The *WNT3* gene may play an active role by counteracting the low activity of the placental Wnt/β-catenin signaling pathway in PE. In addition, *WNT3* gene was also essential in human tooth development, limb development, male fertility, and antidepressant effects [41–44].

In conclusion, we analyzed Wnt/β-catenin signaling pathway-related factors in the placental tissues of women with PE, PB, and TB, and confirmed that the factor with the greatest difference in methylation was the *WNT3* gene. The *WNT3* gene was hypomethylated in PE, and its expression level was significantly higher than that in the other two groups of tissues. The results of in vitro cell studies emphasized that *WNT3* was an activator of signaling pathways. However, signaling pathway activity was reduced in preeclamptic placental tissues. Our results prompted us to speculate that the *WNT3* gene counteracts the low activation state of the Wnt signaling pathway in the preeclamptic placenta through methylation modification. This information deepens researchers’ and clinicians’ understanding of PE pathogenesis. However, the mechanism by which *WNT3* plays its unique role in PE remains unclear and requires further research.

## Supplementary Information

Below is the link to the electronic supplementary material.

Supplementary file1 (PDF 584 kb)

Supplementary file2 (JPG 1909 kb)

Supplementary file3 (JPG 1144 kb)

Supplementary file4 (JPG 1371 kb)

Supplementary file5 (JPG 1506 kb)

Supplementary file6 (JPG 398 kb)

Supplementary file7 (TIF 289 kb)

Supplementary file8 (TIF 121 kb)

Supplementary file9 (TIF 148 kb)

Supplementary file10 (TIF 88 kb)

Supplementary file11 (TIF 152 kb)

Supplementary file12 (TIF 205 kb)

Supplementary file13 (TIF 109 kb)

Supplementary file14 (TIF 124 kb)

Supplementary file15 (TIF 162 kb)

Supplementary file16 (TIF 377 kb)

Supplementary file17 (TIF 225 kb)

Supplementary file18 (TIF 259 kb)

Supplementary file19 (TIF 178 kb)

Supplementary file20 (TIF 172 kb)

Supplementary file21 (TIF 206 kb)

Supplementary file22 (TIF 370 kb)

Supplementary file23 (TIF 196 kb)

Supplementary file24 (TIF 272 kb)

Supplementary file25 (TIF 132 kb)

Supplementary file26 (TIF 114 kb)

Supplementary file27 (TIF 122 kb)

Supplementary file28 (TIF 155 kb)

Supplementary file29 (TIF 201 kb)

## Data Availability

This article is distributed under the terms of the Creative Commons Attribution 4.0 International License (http://creativecommons.org/licenses/by/4.0/), which permits unrestricted use, distribution, and reproduction in any medium, provided you give appropriate credit to the original author(s) and the source, provide a link to the Creative Commons license, and indicate if changes were made. The Creative Commons Public Domain Dedication waiver (http://creativecommons.org/publicdomain/zero/1.0/) applies to the data made available in this article, unless otherwise stated.

## Abbreviations

APC: Adenomatous polyposis coli
CK1: Casein kinase 1
Dvl: Disheveled
DMS: Differential methylated site
EVT: Extravillous trophoblasts
GSK3β: Glycogen synthase kinase 3β
IHC: Immunohistochemistry
LRP: Lipoprotein receptor-related protein
PB: Preterm birth
PCP: Planar cell polarity
PE: Preeclampsia
TB: Term birth
TCF/LEF: T-cell factor/lymphocyte enhancer factor
TSS: Transcription started site
VT: Villous trophoblasts
