# Supplementary material for: WNT3 hypomethylation counteracts low activity of the Wnt signaling pathway in the placenta of preeclampsia
Source: Cell Mol Life Sci. 2021 Oct 4;78(21-22):6995–7008. doi: 10.1007/s00018-021-03941-4 (PMC8558176; doi:10.1007/s00018-021-03941-4)
Supplement: Supplementary file 1 — Supplementary file1 (PDF 584 kb) [file 18_2021_3941_MOESM1_ESM.pdf]

Cellular and Molecular Life Sciences

WNT3 hypomethylation counteracts low activity of the Wnt signaling pathway in the placenta of preeclampsia patients

Zhang Linlin[1*#], Sang Min[1*], Li Ying[1*], Li Yingying[1,4], Yuan Erfeng[1], Yang Lijun[2], Shi Wenli[2]，Yuan Yangyang[3]，Yang bo[3]，Yang Peifeng[2], Yuan Enwu[1]

1. Department of Laboratory Medicine

   Third Affiliated Hospital of Zhengzhou University

   7 Kangfu Qian Street, Zhengzhou City, Henan Province, 450052

   People's Republic of China

2. Department of Obstetrics and Gynecology

   Third Affiliated Hospital of Zhengzhou University

   7 Kangfu Qian Street, Zhengzhou City, Henan Province, 450052

   People's Republic of China

3. Medical Research centre

   Third Affiliated Hospital of Zhengzhou University

   7 Kangfu Qian Street, Zhengzhou City, Henan Province, 450052

   People's Republic of China

4. Marshall medical research centre

   Fifth Affiliated Hospital of Zhengzhou University

   3 Kangfu Qian Street, Zhengzhou City, Henan Province, 450052

   People's Republic of China

***** These authors contributed equally to this work and should be considered co-first authors.

# Corresponding author: Linlin Zhang, M.D., PhD

Associate Professor of Molecular Genetics

Department of Laboratory Medicine

Third Affiliated Hospital of Zhengzhou University

7 Kangfu Qian Street, Zhengzhou City, Henan Province, 450052

People's Republic of China

E-mail: zll7376@zzu.edu.cn

Supplemental Figure

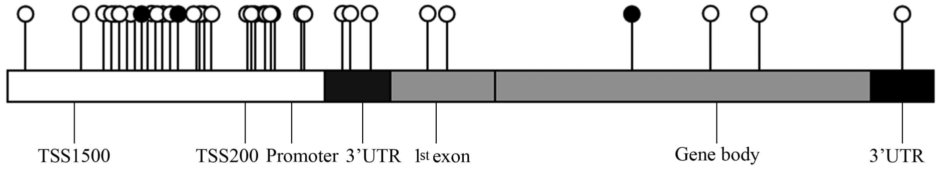

Supplement Fig. 1 Distribution of probes in functional regions of genes. The design of the methylation microarray probes is heterogeneous in Gene functional regions, with the densest probes in the promoter and CpG island regions, and the sparsest probes in the 3'UTR and Gene body regions. TSS: transcription started site.

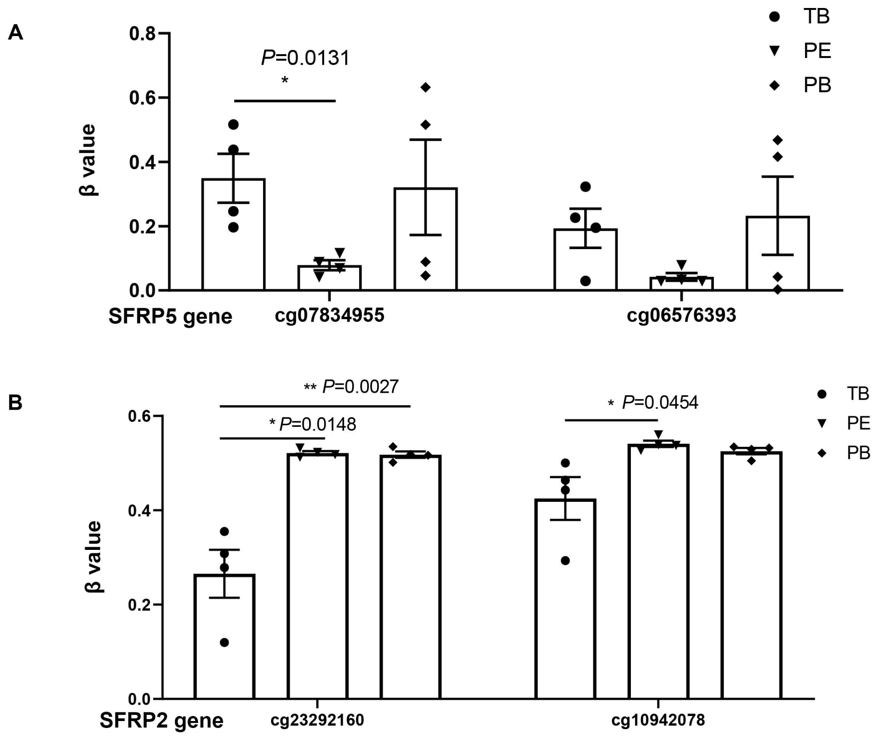

Supplement Fig. 2 The differentially methylated sites of *SFRP5* gene and *SFRP2* gene. Differential methylation sites were selected using $|\Delta\beta| \geq 0.10$ and $P < 0.05$, and the probes located in the body or 3'UTR region were excluded. **A)** The two differential methylation loci of *SFRP5* gene were cg07834955 (located in TSS200) and cg06576393 (located in 1st Exon). These sites showed no statistical significance in the PE and PB groups. **B)** The two differential methylation loci of *SFRP2* gene were cg23292160 (located in TSS200) and cg10942078 (located in TSS1500). These sites showed no statistical significance in the PE and PB groups. (N = 4). Unpaired t test and Welch's t test. GraphPad Prism 8.4.2.

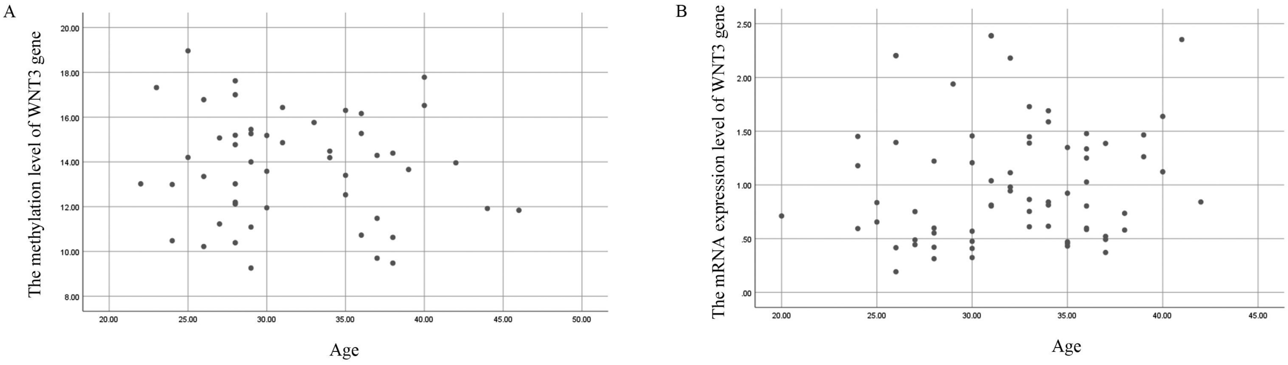

Supplement Fig. 3 Correlation analysis of methylation level (**A**) and mRNA expression level (**B**) of *WNT3* gene with donor age. Statistical analysis of the two pairs of data did not conform to the normal distribution, then spearman correlation analysis found no correlation. IBM SPSS Statistics 25.

Supplemental Table

Supplement Table 1 Significant differential methylation sites in Wnt signaling pathway genes

Gene	ProbeID	TB(β value)	PE(β value)	PB(β value)	Δβ	*P*. value	Location
WNT2B	cg23923856	0.013218056	0.024735337		-0.011517281	0.002381328	1stExon
	cg01257529	0.600626285	0.647974863		-0.047348578	0.026732528	5'UTR
	cg01257529	0.600626285		0.638155888	-0.037529603	0.001189764	5'UTR
	cg17554162	0.71917536		0.759290902	-0.040115541	0.012927018	5'UTR
	cg07360813	0.573779326		0.60454095	-0.030761624	0.045586588	5'UTR
	cg18426113	0.685190655		0.706622903	-0.021432248	0.048920391	5'UTR
	cg11806528		0.154557534	0.179460022	-0.024902488	0.026103821	1stExon
WNT3	cg24114556		0.101896874	0.202725676	-0.100828802	0.033930188	TSS1500
	cg16340130		0.046856661	0.109226146	-0.062369485	0.046149764	Body
	cg02721983		0.643519961	0.670136514	-0.026616553	0.013995921	Body
WNT5B	cg14221149	0.575583957	0.595306857		-0.0197229	0.024295477	5'UTR
	cg09774932	0.583148444	0.614014148		-0.030865705	0.033890516	TSS1500
	cg14221149	0.575583957		0.592384208	-0.016800251	0.019179577	5'UTR
	cg06231565		0.348016011	0.407740939	-0.059724929	0.03557723	TSS200
WNT6	cg10000424	0.013446525	0.020183019		-0.006736493	0.005282092	TSS200
	cg06157334	0.005813548	0.014154131		-0.008340583	0.038849194	TSS200
	cg06157334	0.005813548		0.017515649	-0.011702101	0.001188694	TSS200
WNT8B	cg20462149	0.611830205	0.64358992		-0.031759715	0.032295117	TSS1500
	cg06671996	0.669040759		0.699233873	-0.030193114	0.025562374	TSS1500
WNT9A	cg14343890	0.007292235	0.014318828		-0.007026594	0.014325717	TSS200
	cg14343890	0.007292235		0.016782942	-0.009490707	0.009362508	TSS200
	cg07021033	0.010721925		0.026578526	-0.015856601	0.039096566	TSS1500
WNT10A	cg00821731	0.575000381		0.59536467	-0.020364289	0.024257626	TSS1500

	cg22167208	0.513837652		0.530019181	-0.016181529	0.039422691	TSS1500
WNT10B	cg05164634	0.000905711	0.010705959		-0.009800249	0.037154902	TSS1500
	cg05164634	0.000905711		0.012891772	-0.011986061	0.000564482	TSS1500
	cg18080401	0.066729286		0.086235718	-0.019506432	0.046529959	5'UTR
WNT11	cg17583449	0.083131385	0.111763337		-0.028631952	0.042088621	TSS1500
	cg17583449	0.083131385		0.1337765	-0.050645115	0.006898677	TSS1500
	cg25904812	0.039507383		0.063065387	-0.023558004	0.007057565	TSS1500
WNT16	cg00915831	0.155138429	0.117623231		0.037515199	0.000632914	5'UTR
	cg00915831	0.155138429		0.111219736	0.043918693	0.000476448	5'UTR
	cg14448169		0.046598291	0.038594708	0.008003583	0.03646247	TSS200
SFRP1	cg01495122	0.005053976	0.013393834		-0.008339859	0.006322423	TSS200
	cg01495122	0.005053976		0.012081906	-0.00702793	0.002710701	TSS200
SFRP2	cg23292160	0.26520358	0.521184578		-0.255980998	0.001662264	TSS200
	cg23121156	0.607984442	0.674509474		-0.066525031	0.028700986	TSS200
	cg10942078	0.424840834	0.540652473		-0.115811639	0.037971625	TSS1500
	cg23292160	0.26520358		0.517679059	-0.252475479	0.001850033	TSS200
	cg10663078	0.516463075		0.546066937	-0.029603862	0.005982826	TSS1500
	cg25775322	0.529147949		0.577397263	-0.048249313	0.00854188	TSS200
SFRP5	cg16536739	0.010742512	0.001705883		0.009036629	0.005907209	TSS1500
	cg07834955	0.349195243	0.078647577		0.270547666	0.010095993	TSS200
	cg06576393	0.193451662	0.04230132		0.151150342	0.04288469	1[st]Exon
DKK3	cg16915821	0.005453232	0.01594791		-0.010494679	0.008713791	TSS200
	cg06188670	0.011079074		0.021549783	-0.010470709	0.010141651	TSS200
	cg22082397		0.023699299	0.033310422	-0.009611123	0.018381408	TSS1500
	cg04100696		0.023968755	0.011343162	0.012625593	0.037739575	TSS200
	cg18737956		0.568911011	0.546904358	0.022006653	0.049896078	TSS1500
DDK4	cg09297903	0.592525872		0.605339585	-0.012813713	0.044548592	TSS200

	cg06899970	0.233180436		0.120214837	0.112965599	0.048594228	5'UTR
WIF1	cg03509412	0.513133705	0.545153497		-0.032019792	0.001187279	5'UTR

TB, term birth; PE, preeclampsia; PB, preterm birth. TSS: transcription started site

Most probes of *WNT1*, *WNT2*, *WNT3A*, *WNT4*, *WNT5A*, *WNT7A*, *WNT7B*, *WNT8A*, *WNT9B*, *SFRP4*, *DKK1*, and *DKK2* were located in the body or 3'UTR region, while there was no significant difference in the methylation of probes located in the transcription promoter region ($P > 0.05$).
