# Supplementary figures and images for: WNT3 hypomethylation counteracts low activity of the Wnt signaling pathway in the placenta of preeclampsia
Source: Cell Mol Life Sci. 2021 Oct 4;78(21-22):6995–7008. doi: 10.1007/s00018-021-03941-4 (PMC8558176; doi:10.1007/s00018-021-03941-4)

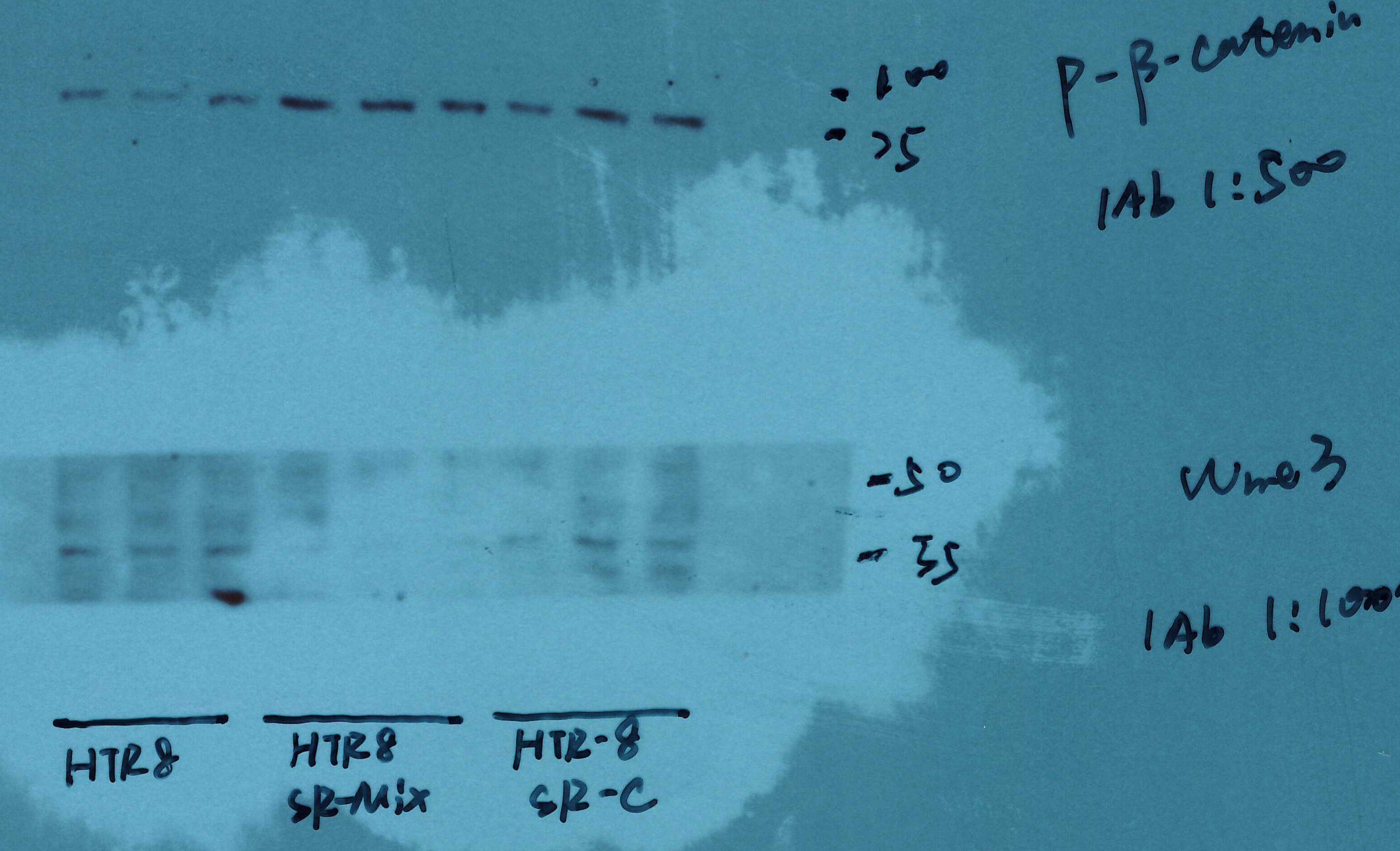

Supplement: Supplementary file 2 — Supplementary file2 (JPG 1909 kb) [file 18_2021_3941_MOESM2_ESM.jpg]

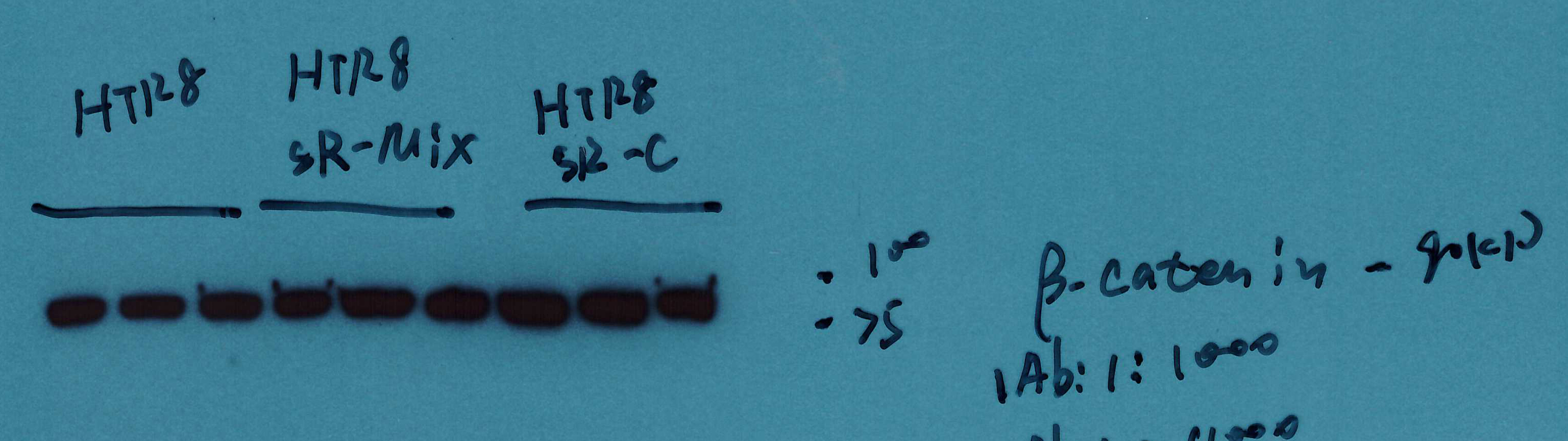

Supplement: Supplementary file 3 — Supplementary file3 (JPG 1144 kb) [file 18_2021_3941_MOESM3_ESM.jpg]

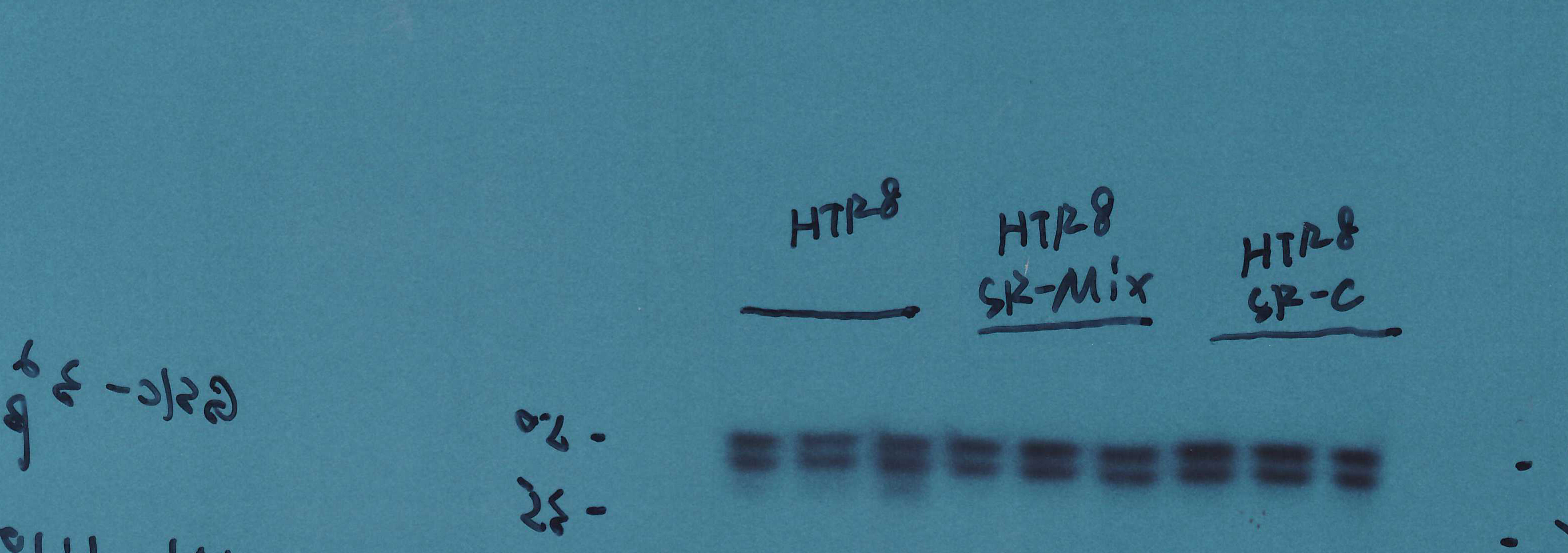

Supplement: Supplementary file 4 — Supplementary file4 (JPG 1371 kb) [file 18_2021_3941_MOESM4_ESM.jpg]

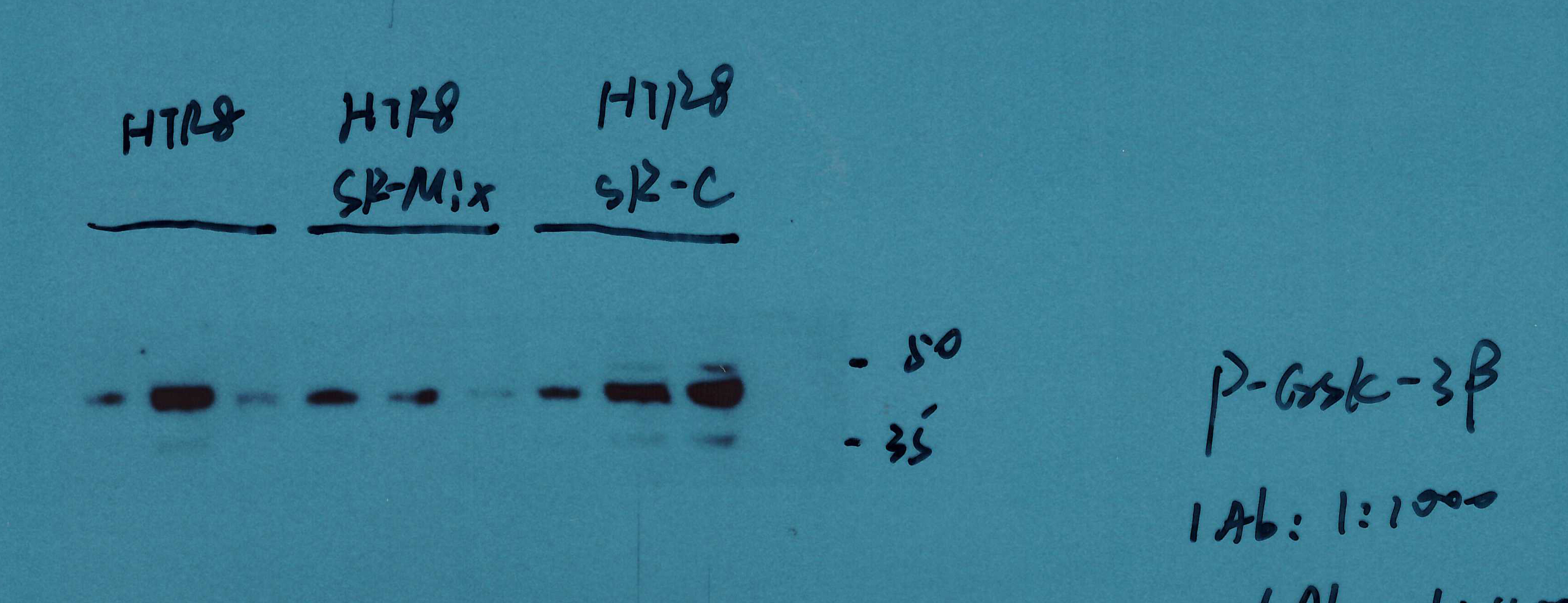

Supplement: Supplementary file 5 — Supplementary file5 (JPG 1506 kb) [file 18_2021_3941_MOESM5_ESM.jpg]

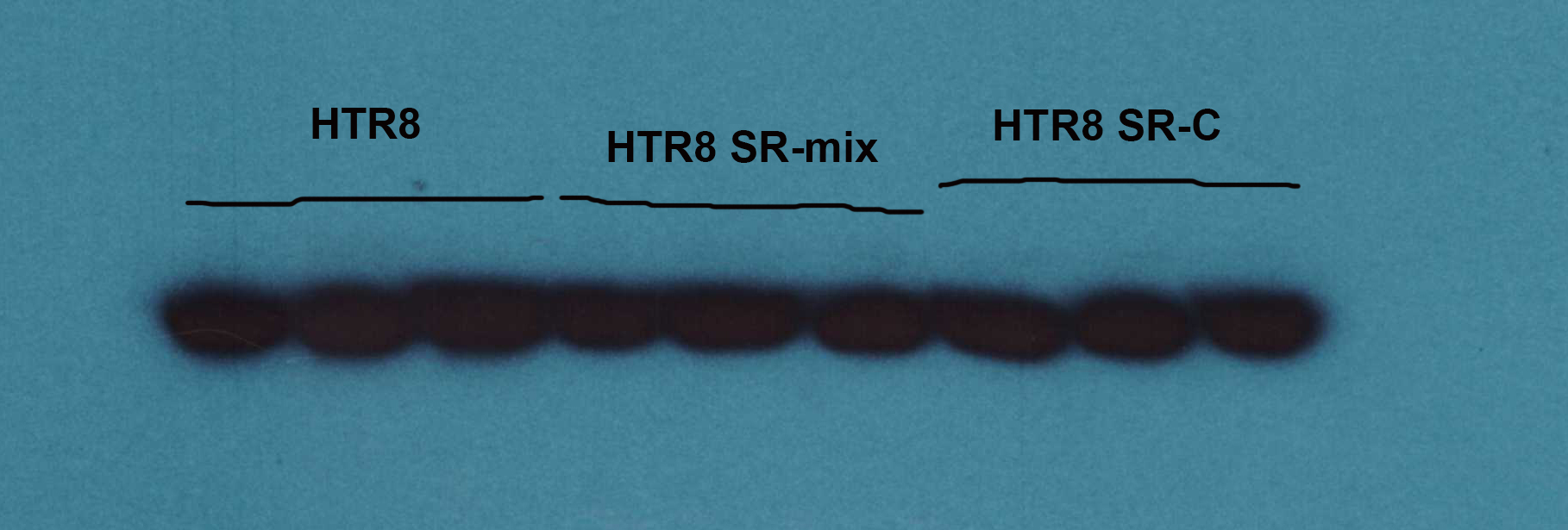

Supplement: Supplementary file 6 — Supplementary file6 (JPG 398 kb) [file 18_2021_3941_MOESM6_ESM.jpg]

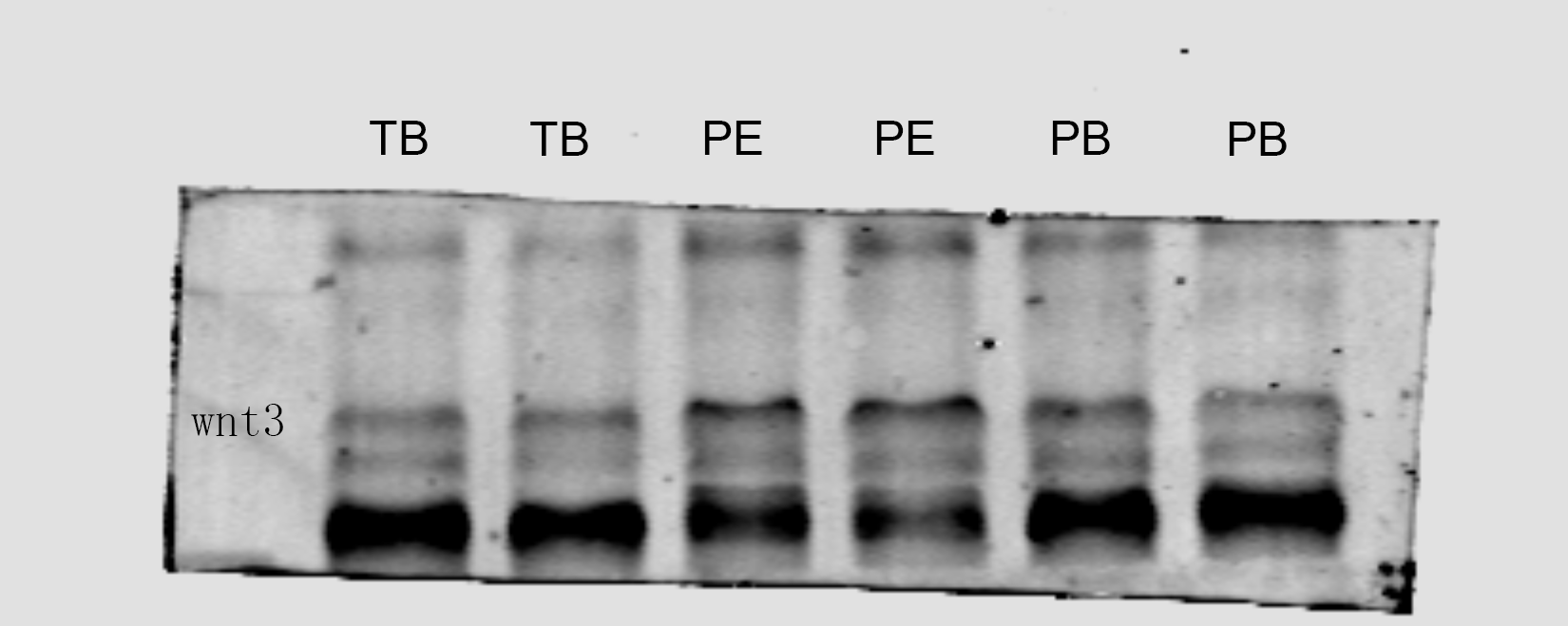

Supplement: Supplementary file 7 — Supplementary file7 (TIF 289 kb) [file 18_2021_3941_MOESM7_ESM.tif]

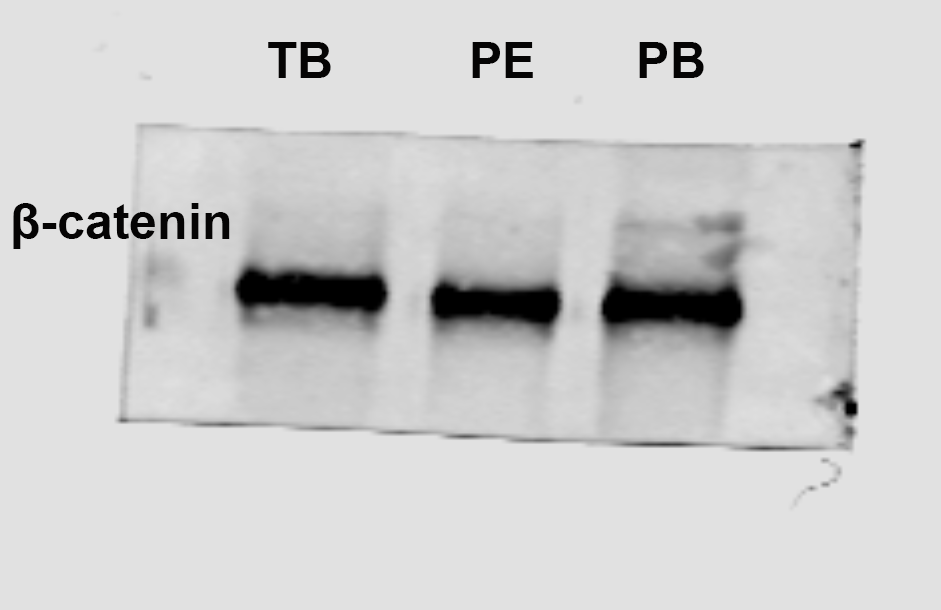

Supplement: Supplementary file 8 — Supplementary file8 (TIF 121 kb) [file 18_2021_3941_MOESM8_ESM.tif]

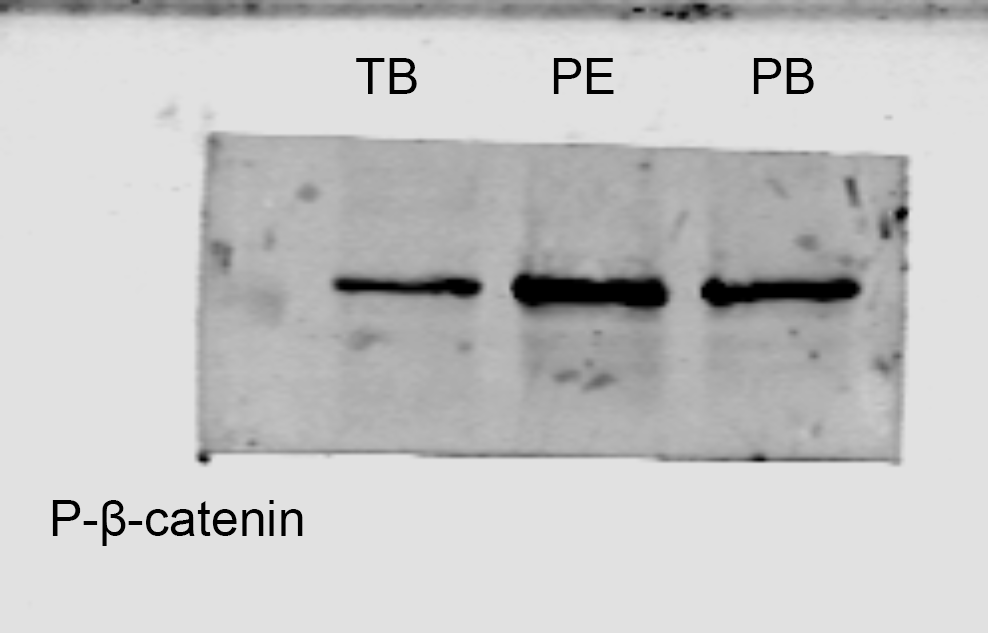

Supplement: Supplementary file 9 — Supplementary file9 (TIF 148 kb) [file 18_2021_3941_MOESM9_ESM.tif]

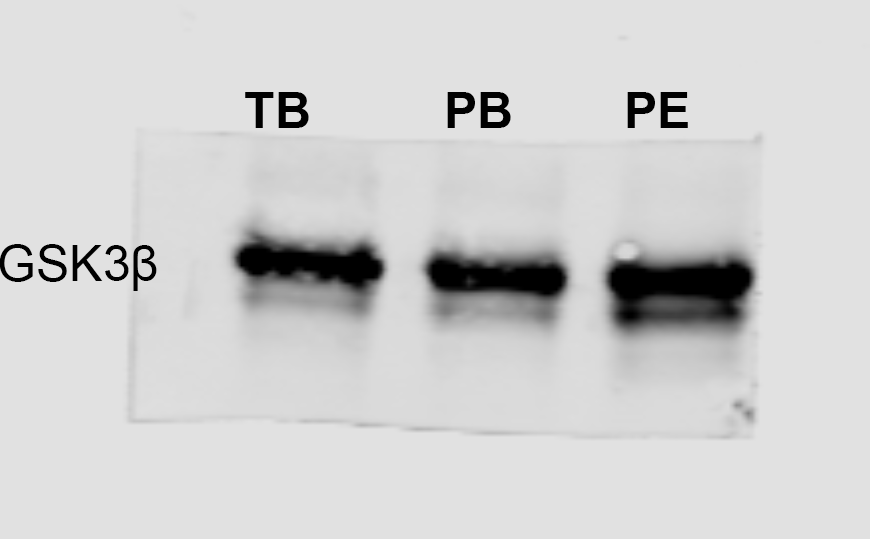

Supplement: Supplementary file 10 — Supplementary file10 (TIF 88 kb) [file 18_2021_3941_MOESM10_ESM.tif]

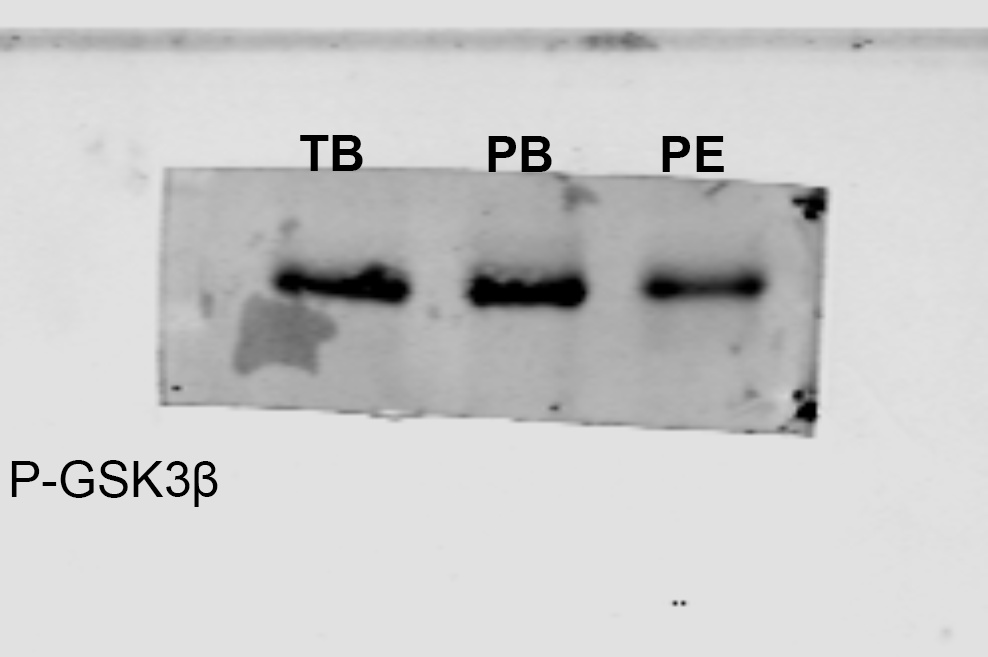

Supplement: Supplementary file 11 — Supplementary file11 (TIF 152 kb) [file 18_2021_3941_MOESM11_ESM.tif]

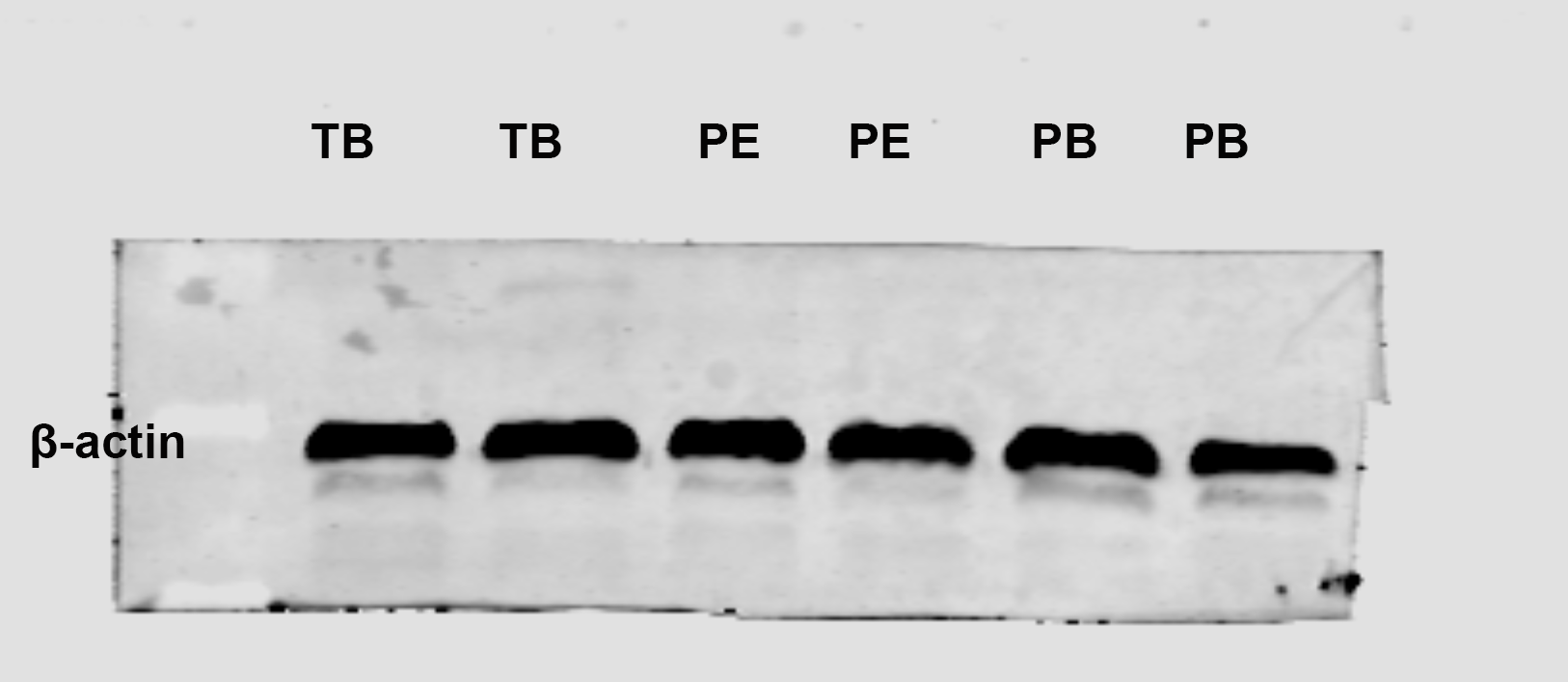

Supplement: Supplementary file 12 — Supplementary file12 (TIF 205 kb) [file 18_2021_3941_MOESM12_ESM.tif]

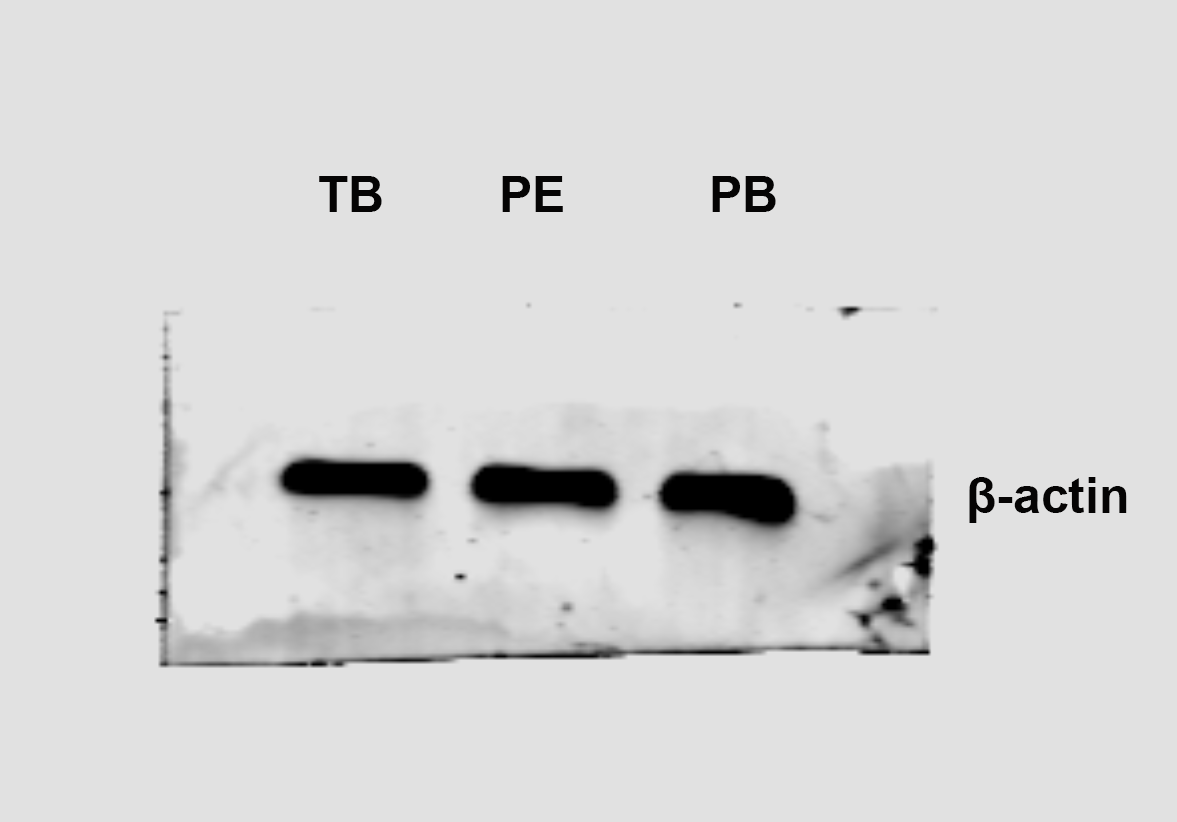

Supplement: Supplementary file 13 — Supplementary file13 (TIF 109 kb) [file 18_2021_3941_MOESM13_ESM.tif]

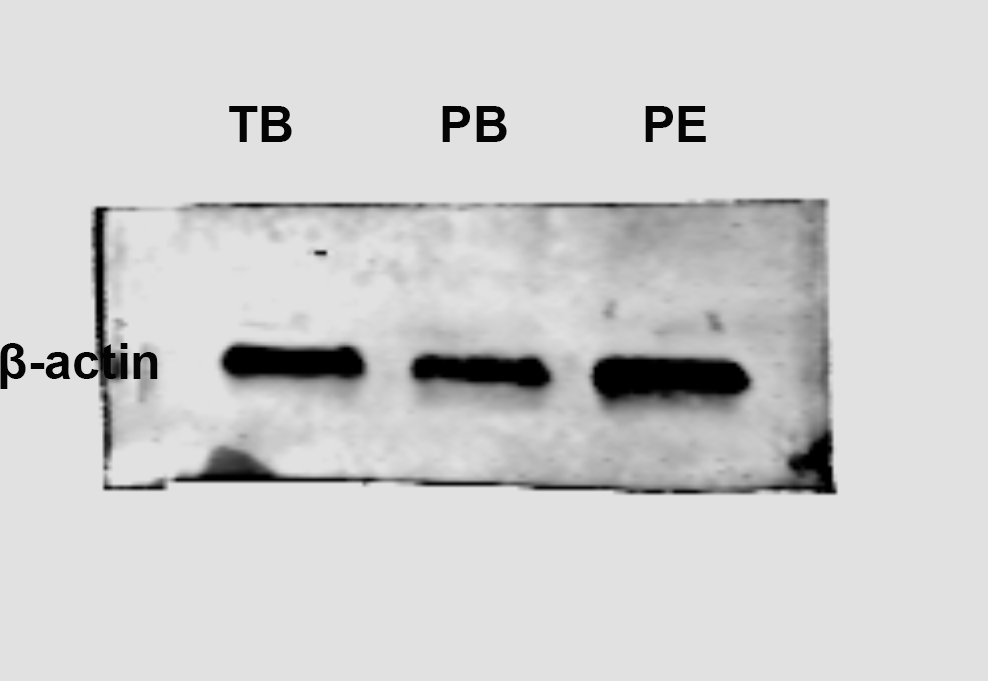

Supplement: Supplementary file 14 — Supplementary file14 (TIF 124 kb) [file 18_2021_3941_MOESM14_ESM.tif]

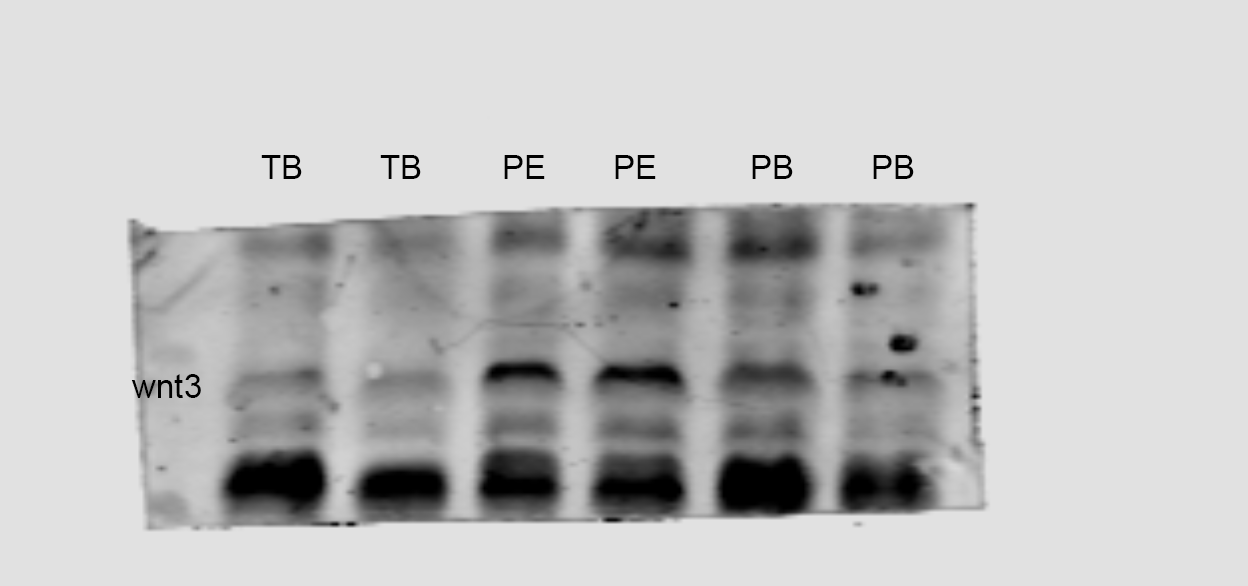

Supplement: Supplementary file 15 — Supplementary file15 (TIF 162 kb) [file 18_2021_3941_MOESM15_ESM.tif]

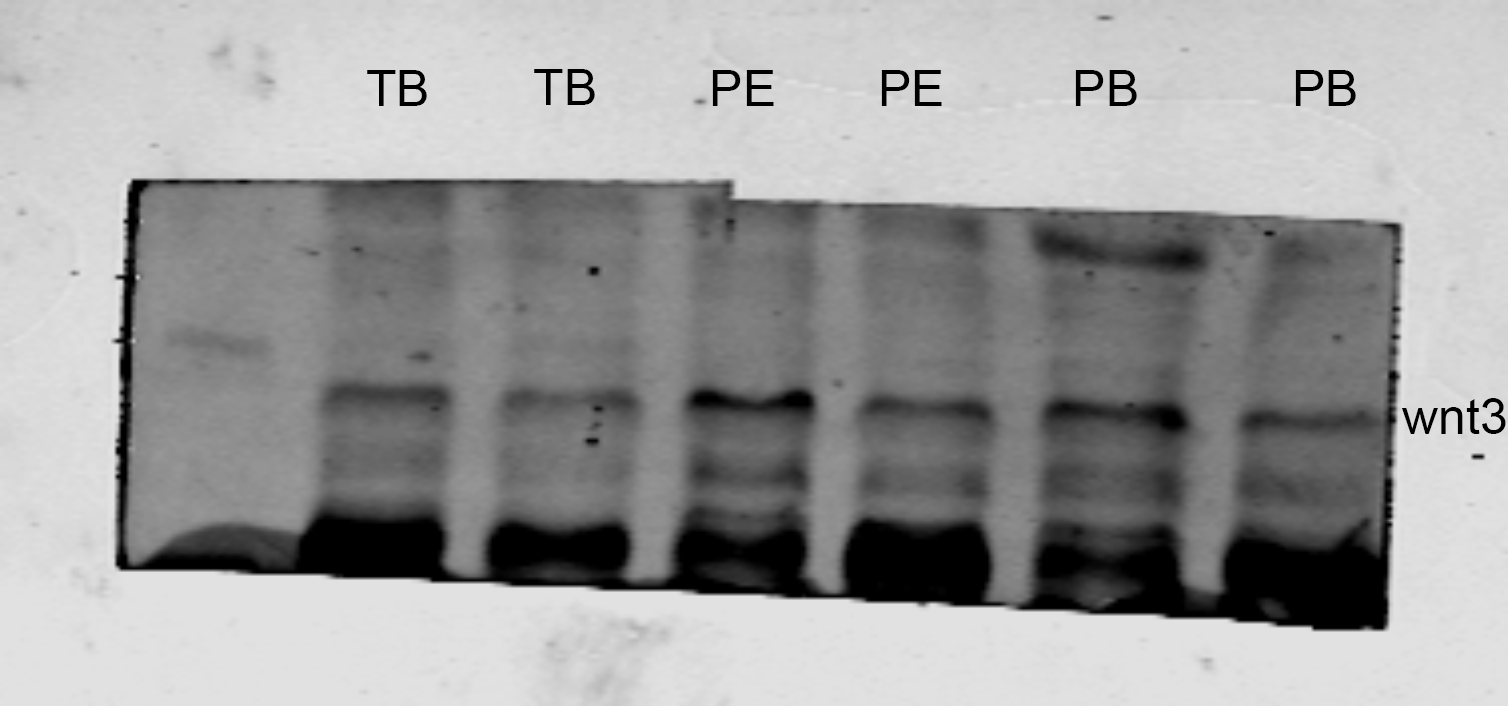

Supplement: Supplementary file 16 — Supplementary file16 (TIF 377 kb) [file 18_2021_3941_MOESM16_ESM.tif]

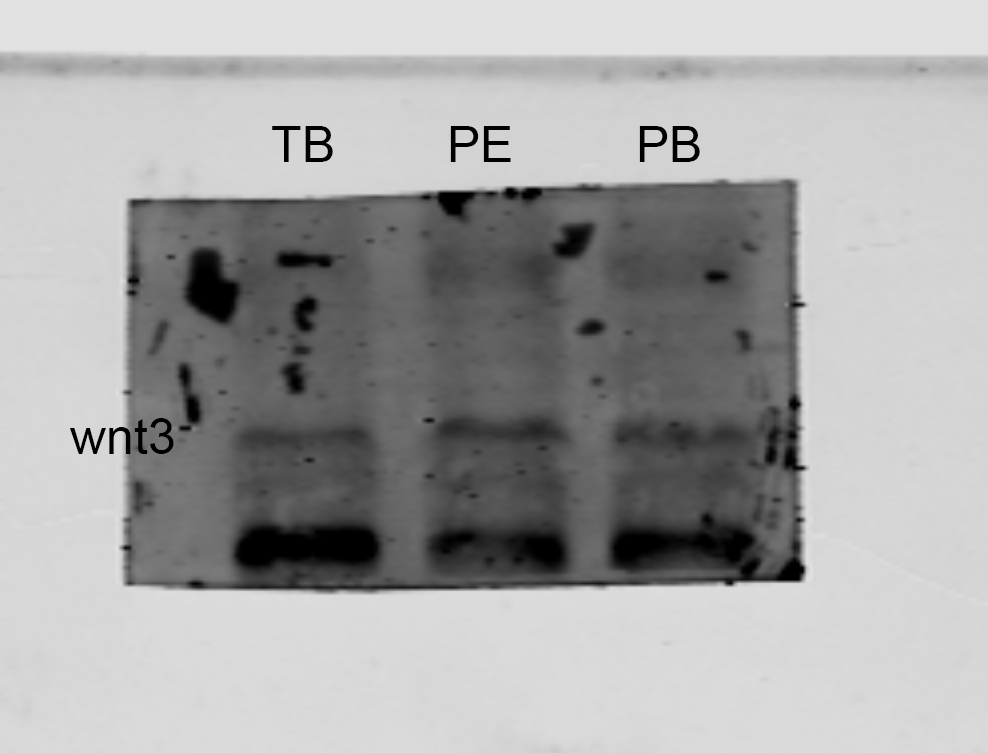

Supplement: Supplementary file 17 — Supplementary file17 (TIF 225 kb) [file 18_2021_3941_MOESM17_ESM.tif]

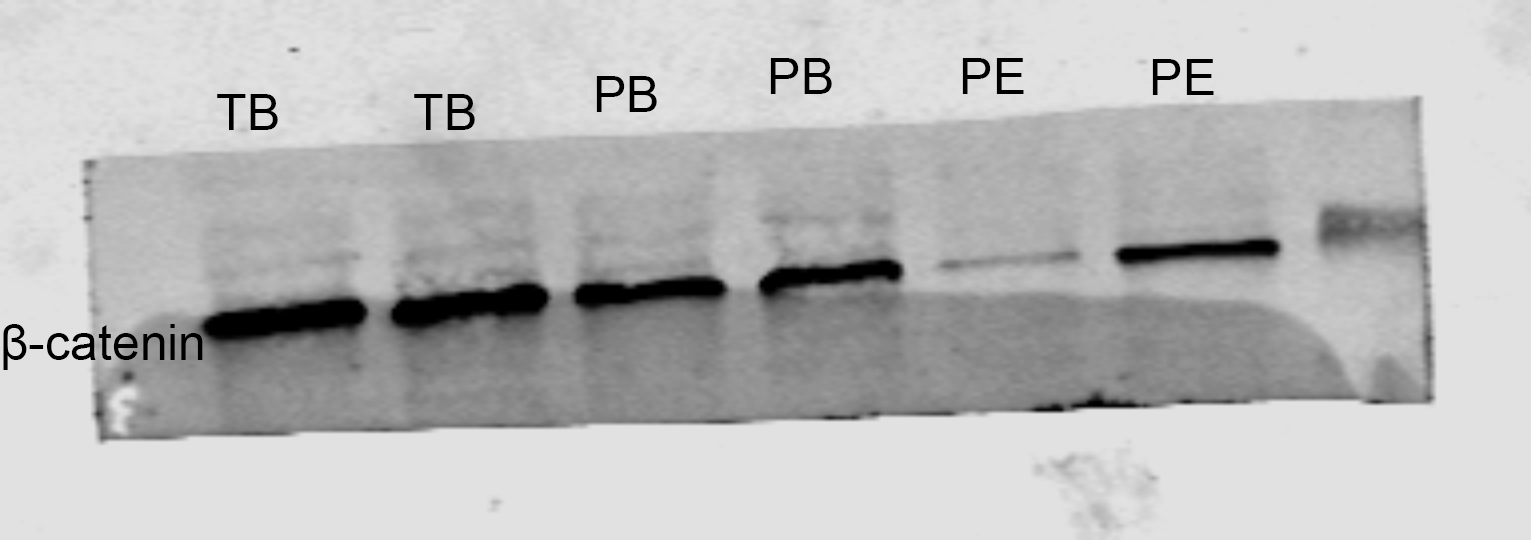

Supplement: Supplementary file 18 — Supplementary file18 (TIF 259 kb) [file 18_2021_3941_MOESM18_ESM.tif]

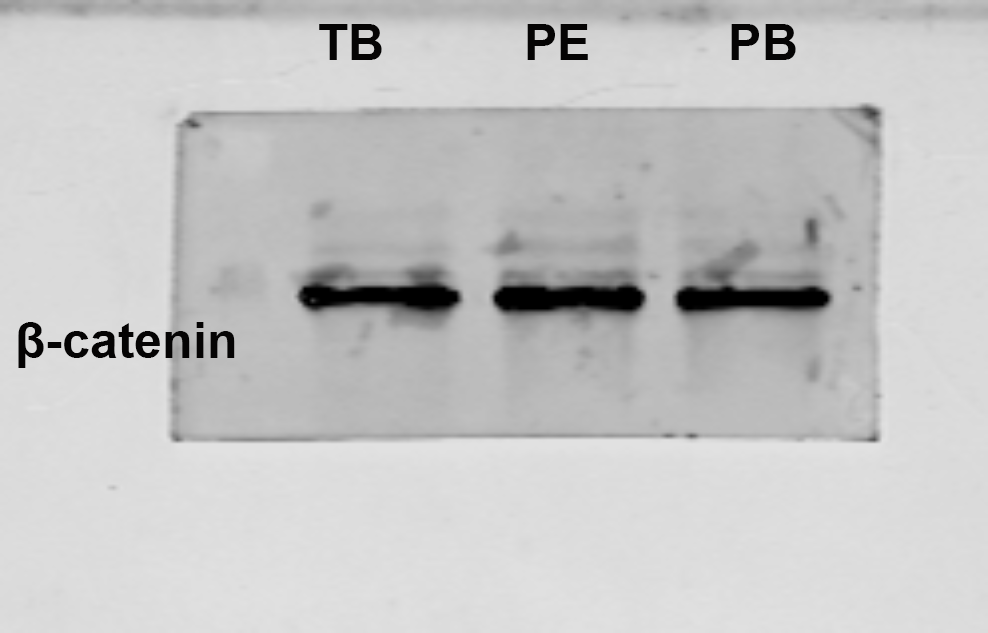

Supplement: Supplementary file 19 — Supplementary file19 (TIF 178 kb) [file 18_2021_3941_MOESM19_ESM.tif]

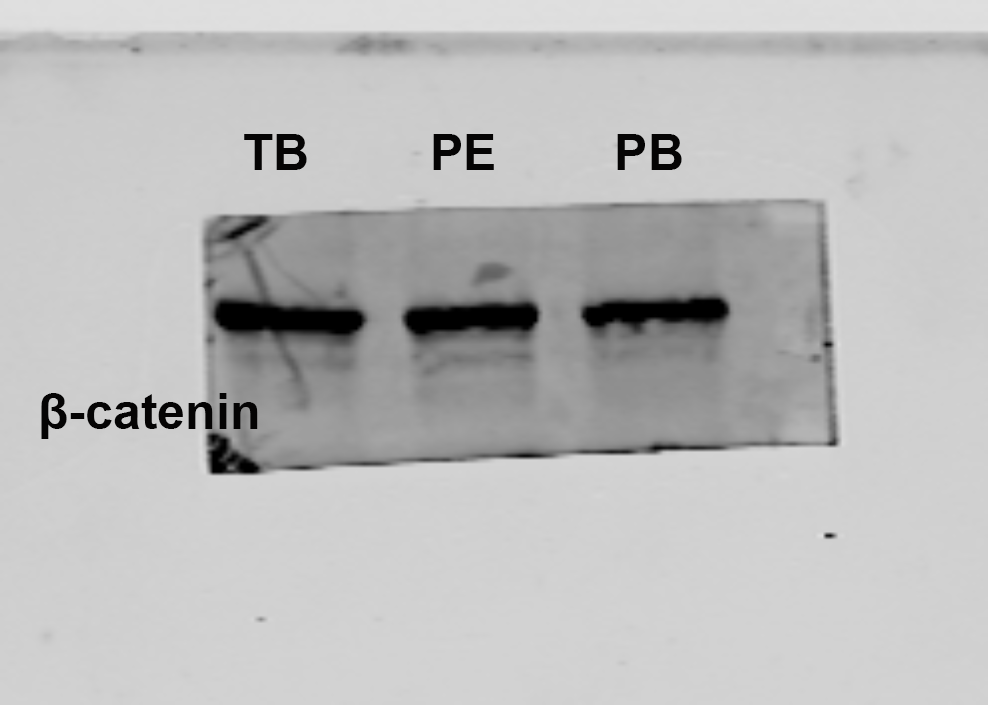

Supplement: Supplementary file 20 — Supplementary file20 (TIF 172 kb) [file 18_2021_3941_MOESM20_ESM.tif]

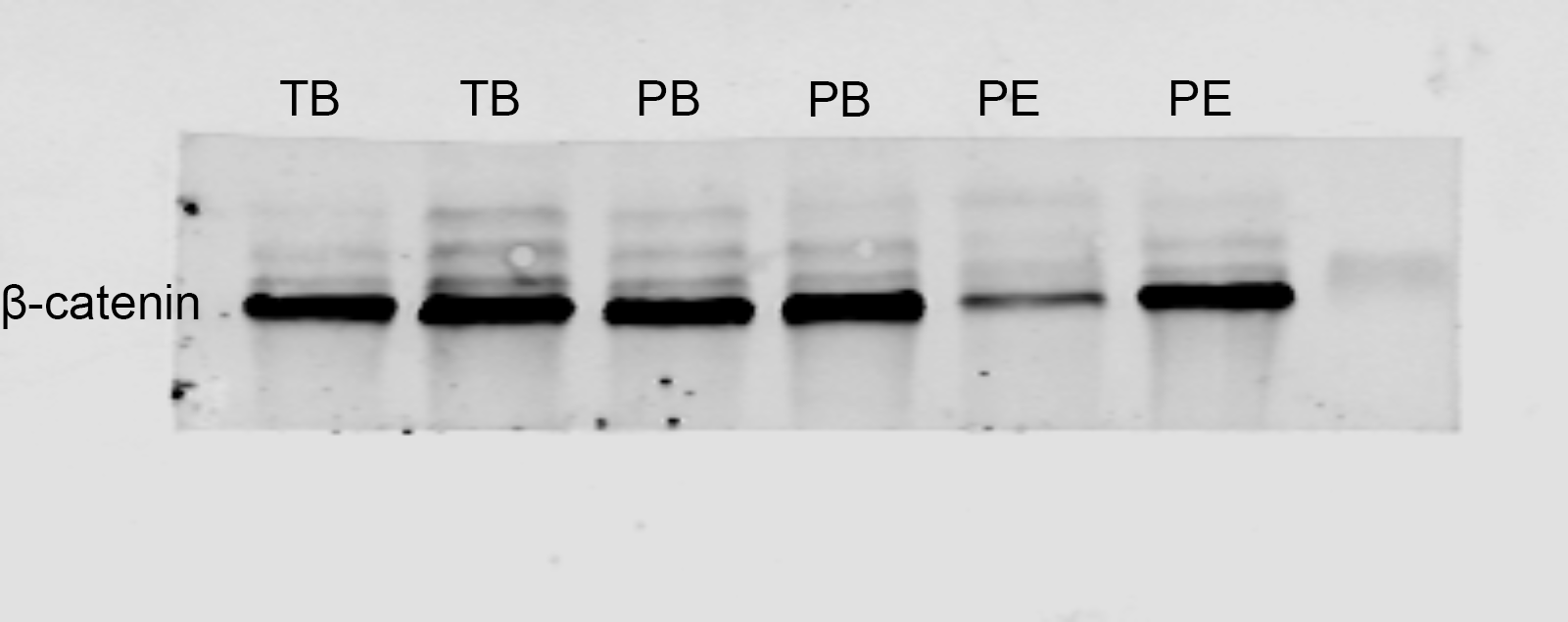

Supplement: Supplementary file 21 — Supplementary file21 (TIF 206 kb) [file 18_2021_3941_MOESM21_ESM.tif]

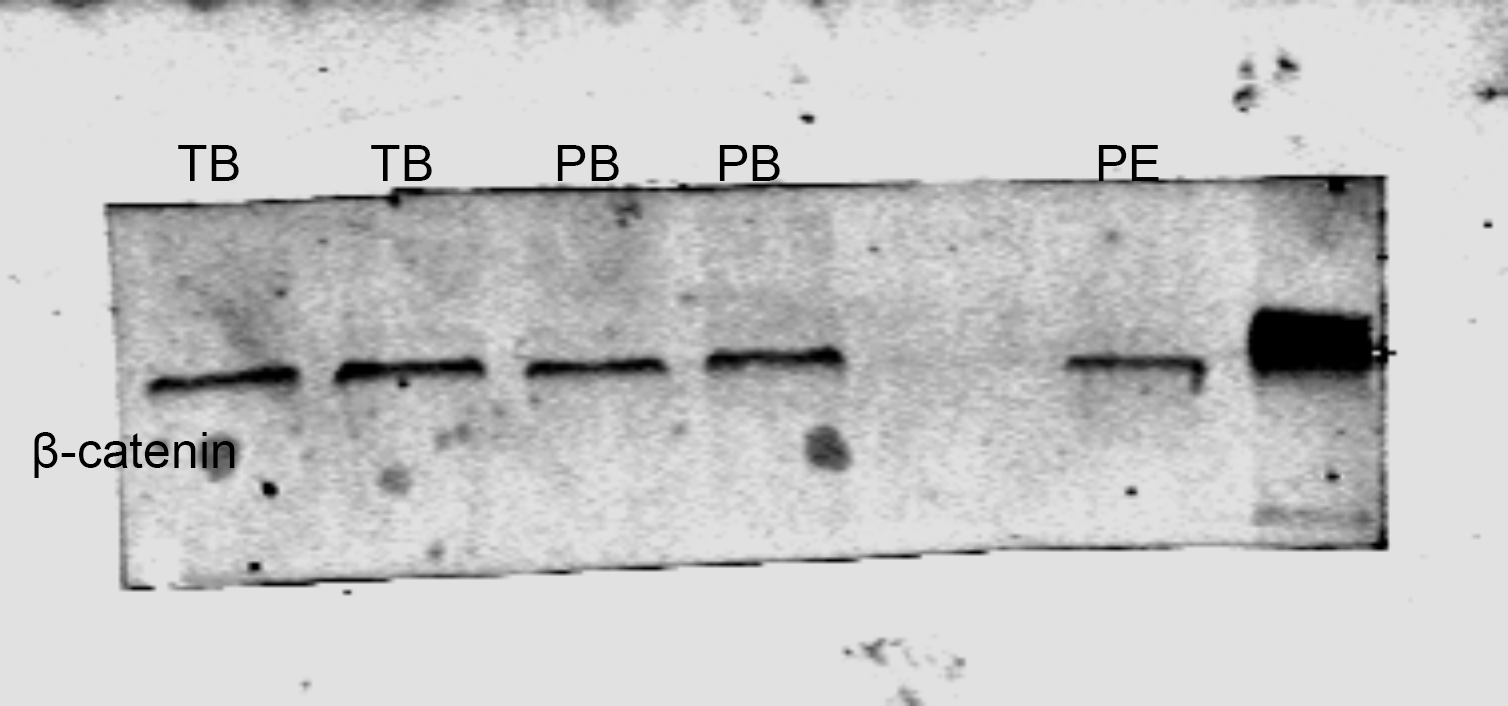

Supplement: Supplementary file 22 — Supplementary file22 (TIF 370 kb) [file 18_2021_3941_MOESM22_ESM.tif]

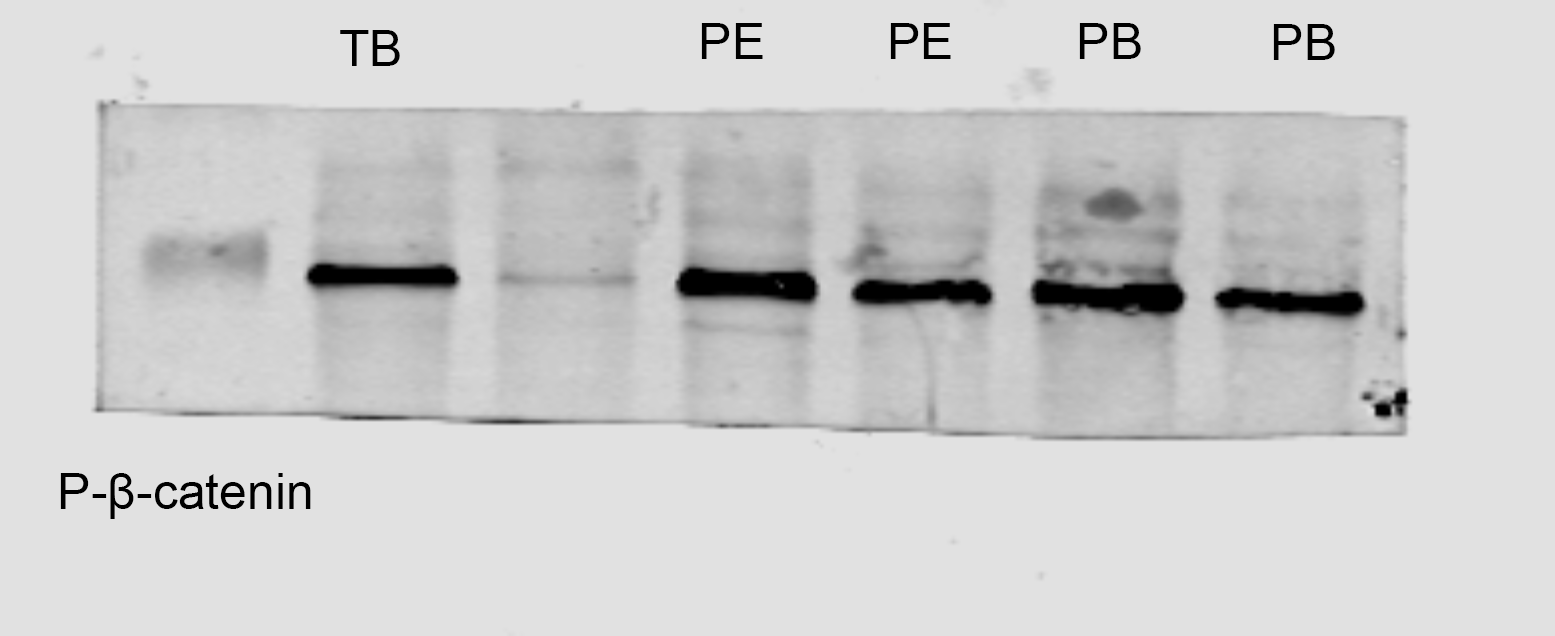

Supplement: Supplementary file 23 — Supplementary file23 (TIF 196 kb) [file 18_2021_3941_MOESM23_ESM.tif]

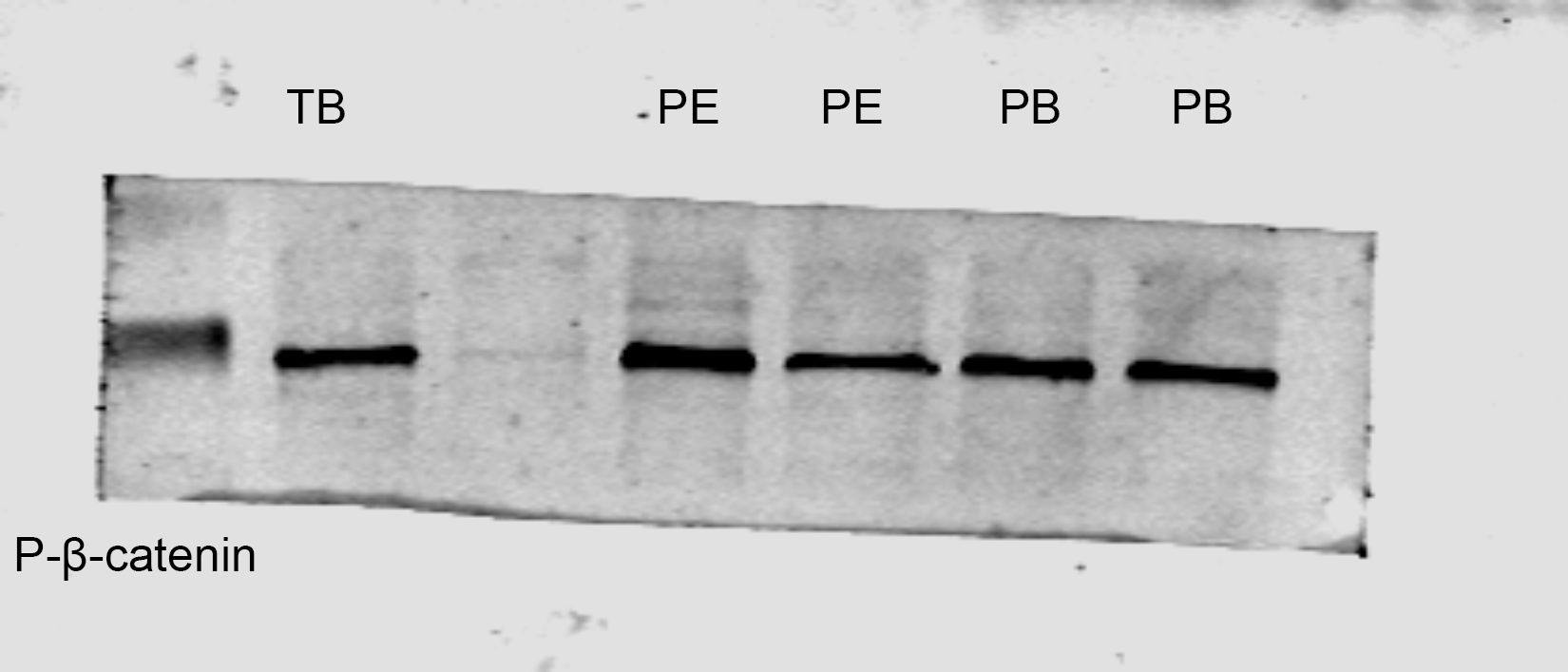

Supplement: Supplementary file 24 — Supplementary file24 (TIF 272 kb) [file 18_2021_3941_MOESM24_ESM.tif]

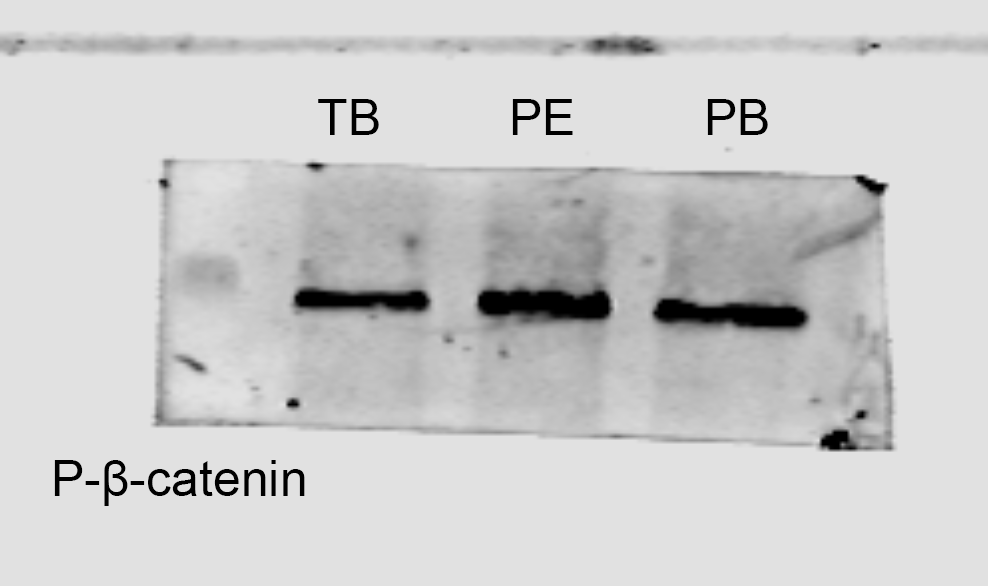

Supplement: Supplementary file 25 — Supplementary file25 (TIF 132 kb) [file 18_2021_3941_MOESM25_ESM.tif]

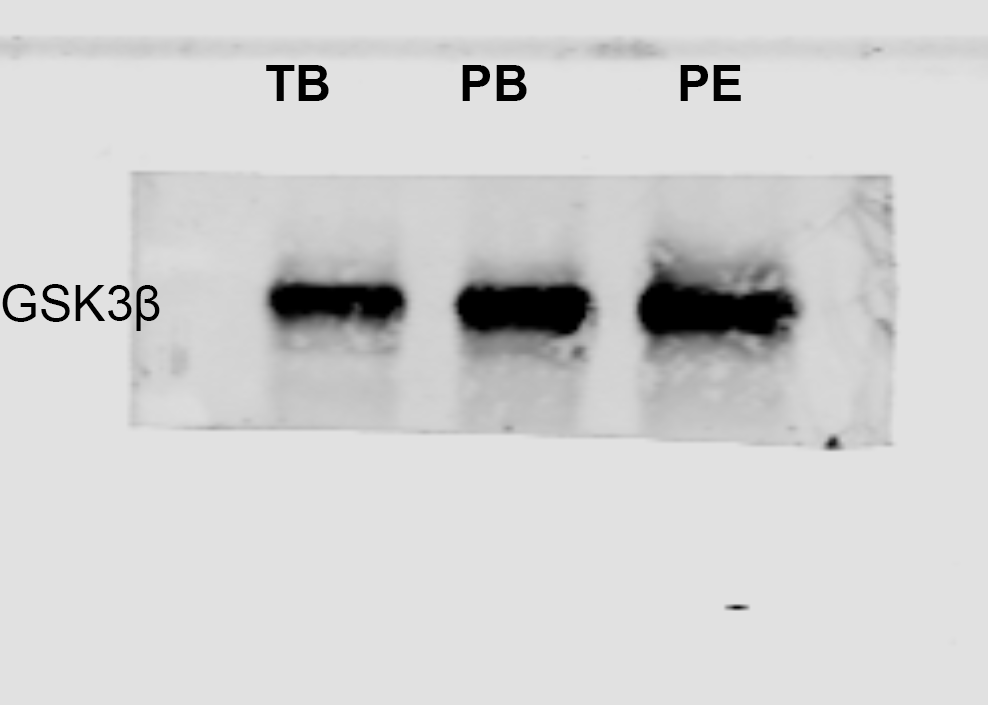

Supplement: Supplementary file 26 — Supplementary file26 (TIF 114 kb) [file 18_2021_3941_MOESM26_ESM.tif]

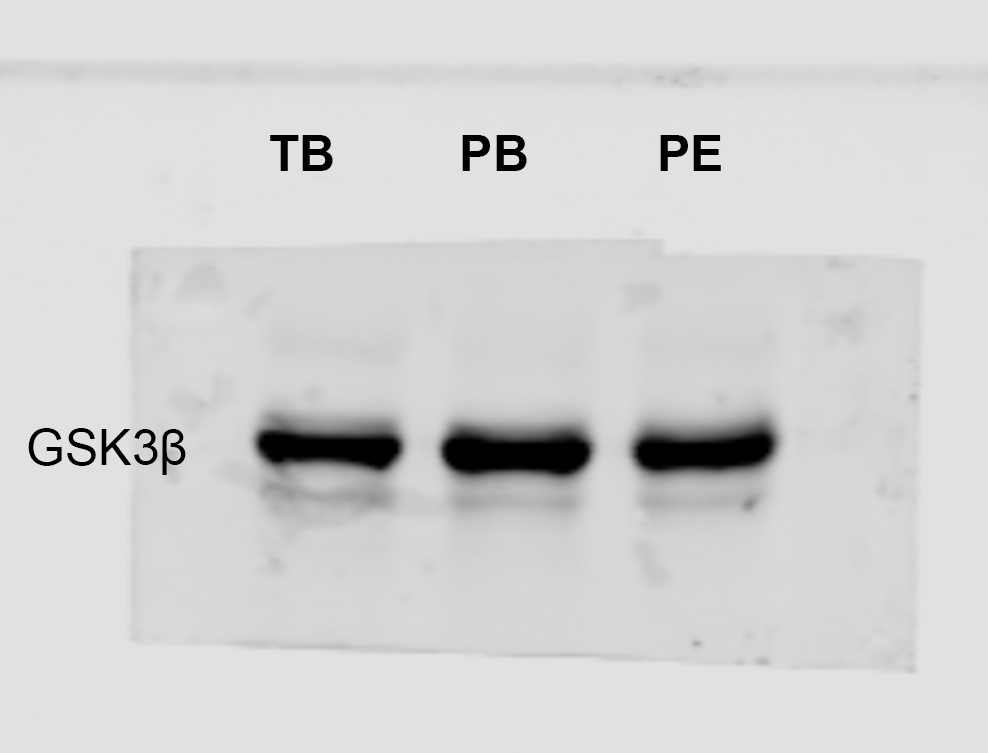

Supplement: Supplementary file 27 — Supplementary file27 (TIF 122 kb) [file 18_2021_3941_MOESM27_ESM.tif]

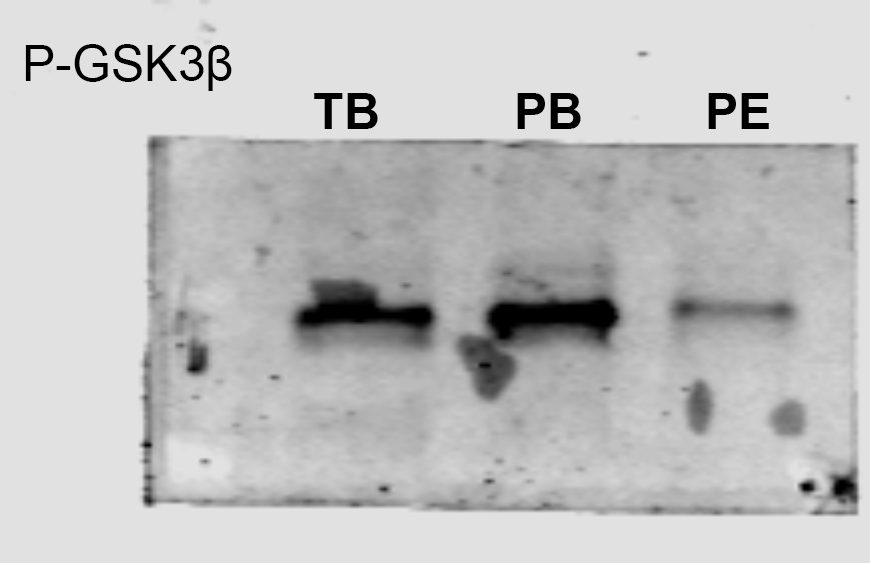

Supplement: Supplementary file 28 — Supplementary file28 (TIF 155 kb) [file 18_2021_3941_MOESM28_ESM.tif]

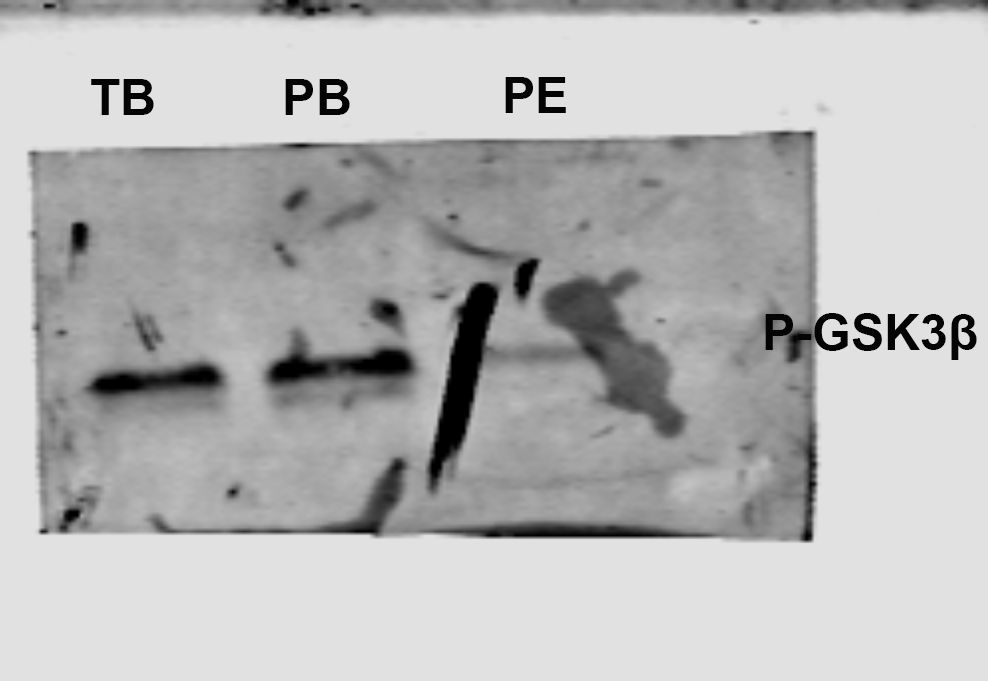

Supplement: Supplementary file 29 — Supplementary file29 (TIF 201 kb) [file 18_2021_3941_MOESM29_ESM.tif]
